# What is new with Artificial Intelligence? Human–agent interactions through the lens of social agency

**DOI:** 10.3389/fpsyg.2022.954444

**Published:** 2022-09-29

**Authors:** Marine Pagliari, Valérian Chambon, Bruno Berberian

**Affiliations:** ^1^Institut Jean Nicod, Département d’Études Cognitives, École Normale Supérieure, Centre National de la Recherche Scientifique, Paris Sciences et Lettres University, Paris, France; ^2^Information Processing and Systems, Office National d’Etudes et Recherches Aérospatiales, Salon de Provence, France

**Keywords:** sense of agency, social agency, joint action, explainable Artificial Intelligence, acceptability, human–AI interaction

## Abstract

In this article, we suggest that the study of social interactions and the development of a “sense of agency” in joint action can help determine the content of relevant explanations to be implemented in artificial systems to make them “explainable.” The introduction of automated systems, and more broadly of Artificial Intelligence (AI), into many domains has profoundly changed the nature of human activity, as well as the subjective experience that agents have of their own actions and their consequences – an experience that is commonly referred to as sense of agency. We propose to examine the empirical evidence supporting this impact of automation on individuals’ sense of agency, and hence on measures as diverse as operator performance, system explicability and acceptability. Because of some of its key characteristics, AI occupies a special status in the artificial systems landscape. We suggest that this status prompts us to reconsider human–AI interactions in the light of human–human relations. We approach the study of joint actions in human social interactions to deduce what key features are necessary for the development of a reliable sense of agency in a social context and suggest that such framework can help define what constitutes a good explanation. Finally, we propose possible directions to improve human–AI interactions and, in particular, to restore the sense of agency of human operators, improve their confidence in the decisions made by artificial agents, and increase the acceptability of such agents.

## Introduction

Over the past few decades, automation has profoundly changed our daily lives, and predictions for the future indicate that this trend will continue to grow, especially with the advent of Artificial Intelligence (AI). The introduction of automation into our lives has many benefits, including increased safety for humans. However, the interposition of automated systems between human operators and control processes has radically changed the nature of human activity, from direct manual control to partial or full supervision. While the negative impact of such a change on operators’ performance has been widely documented, the cognitive mechanisms involved in this degradation are still poorly understood. We suggest that studying social interactions and the development of a sense of agency in human joint actions can help refine the content of explanations to be implemented in AI to improve human–AI interactions.

The sense of agency (i.e., the feeling of being at the origin of and controlling our actions and their consequences on the environment) has been presented as a potential mediator of this degraded state of performance. Indeed, interaction with an automated system can particularly alter the experience of control over our actions and create ambiguous situations as to who has control. The detrimental effect of automation on agency has been documented in a large number of studies. From an operational point of view, loss of agency in human–machine interactions leads not only to a decrease in the acceptability of automated systems, but can also lead to a disengagement of the operator from the task at hand. The continuing evolution of automated systems, and in particular the advent of AI, is likely to further alter the relationship between agency and technology. We propose to investigate the nature of this relatively recent change in human technological history and its potential impact on the agentive experience of human operators.

To do so, we will first examine whether, and to what extent, some of the negative consequences of using technology can be explained by a disruption in the development of our sense of agency. We will discuss how the experience of agency develops when we act alone or in interaction with automated systems, and then highlight the singularity of AI compared to automated systems in general, and the new questions it raises in terms of interaction with human operators. We will examine some key features of AI that contribute to changing the role of the technological tool in human decision-making processes and their impact on the sense of agency. We will see how these changes invite to consider human–AI interactions in the light of human–human relations. We will then turn to studies of joint action in social interactions in an attempt to deduce the characteristics that enable the development of a sense of agency in social contexts. Finally, we will propose to transfer insights gained from our study of human social agency to the field of eXplainable Artificial Intelligence (XAI). This research area aims at making AI more readable and transparent to humans by producing explanations of how AI makes decisions. Giving access to the different levels of intention (proximal and distal) implemented by AI could help restore human operators’ sense of agency, improve their confidence in the decisions made by artificial agents, and ultimately increase acceptability toward such agents.

## Sense of agency and technology

### Impact of automation on human operators

Over the past few decades, automation technology has become a pervasive phenomenon and has profoundly changed our daily lives. Automation refers to the process of partially or completely handling over a task usually performed by a human to a machine or system (Sheridan, 2002). The initial reason for the introduction of automation was to reduce the workload of operators, and thus reduce operational costs and errors, while increasing accuracy (Sarter et al., 1997). In aviation, the introduction of automated technologies, such as systems that provide vital automated aids, has significantly improved current levels of safety (International Civil Aviation Organization, 2017).

The complexity and ingenuity that characterize automated systems tend to focus public attention on the technical capabilities of automation. However, while the introduction of automation into a system can be viewed as a simple substitution of a machine activity for a human activity (Woods and Tinapple, 1999), human activities are impacted often unintentionally and unanticipated by automation designers. As a matter of fact, the interposition of automated systems between human operators and control processes has radically changed the nature of human activity from direct manual control to partial or total supervision (Dekker and Woods, 2002). This change is far from trivial and creates new burdens and complexities for individuals and teams of practitioners charged with operating, troubleshooting, and managing automated systems.

The negative impact of such a change on operators activities is widely documented through the notion of *out-of-the-loop performance problem*, i.e., OOTL (Endsley and Kiris, 1995; Kaber and Endsley, 1997). An out-of-the-control-loop operator has difficulty detecting errors or failures in the system (Ephrath and Young, 1981; Kessel and Wickens, 1982), understanding its current state (Sarter and Woods, 1995; Sarter et al., 1997; Christoffersen and Woods, 2002), and determining appropriate actions for the next task (Endsley, 1999). Decreased vigilance, complacency or overconfidence in the system’s capabilities, and a loss of situational awareness on the part of the operator have been identified as factors that may contribute to this phenomenon. For decades, the problem of OOTL performance has been a major concern in the human factor literature. However, the cognitive mechanisms involved in the OOTL phenomenon are still poorly understood.

Recently, the notion of agency has been presented as a potential mediator of this degraded state of performance (Berberian, 2019). The term ‘sense of agency’ refers to the subjective awareness of initiating, executing, and controlling one’s own volitional actions in the world (Jeannerod, 2003). This form of self-awareness is important not only for motor control but also for social interactions, the ascription of causal responsibility, and serves as a key motivational force for human behavior.

Early studies in the aerospace field sought to understand how automation in aviation affects the sense of control. Automation was first shown to reduce pilots’ sense of control (Norman et al., 1990). More recently, Berberian et al. (2012) explored the modulation of the sense of agency through automation in a task implementing different degrees of autopilot assistance in a flight simulator. In this study, subjective reports of sense of agency were asked of participants to measure the degree to which they felt they had caused the maneuver to avoid the conflict, ranging from no causal involvement to high causal involvement. The results showed that participants’ sense of agency decreased with the level of automation involved (Berberian et al., 2012). This alteration of the agentive experience has since been highlighted in various works (Yun et al., 2018; Grynszpan et al., 2019; Sahaï et al., 2019; Zanatto et al., 2021). In contrast, other research suggests a positive influence of automation on the sense of agency (Wen et al., 2015a,^2021^; Vantrepotte et al., 2022). As an illustration, Wen et al. (2015a) designed a computer assistance program in a dot-moving game, in which the computer only ignored participants’ incorrect commands. The results showed that when there is a response delay in the game, making the task very difficult, the computer assistance significantly increased participants’ sense of agency compared to the condition where all participants’ commands were executed (Wen et al., 2015a).

**FIGURE 1:**
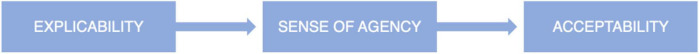
Link between explicability, sense of agency, and acceptability. We suggest that the presence of explanations for the system decision increases the user’s sense of agency, which in turn increases the acceptability of the system itself.

Taken together, these results show that advances in automation technologies can modulate the development of the experience of agency. This modulation of our experience of agency can have dramatic operational consequences along a number of key dimensions:

- *Experience of agency and acceptability:* Decrease in the sense of agency when interacting with highly automated systems is likely to seriously threaten the acceptability of the system’s decisions by human operators. To be acceptable, a new technology must be reliable, efficient, and useful. However, these qualities do not guarantee acceptability by the human operator. Indeed, users strongly desire to feel that “they are in charge of the system” and that the system “responds to their actions” (Shneiderman and Plaisant, 2004). Moreover, a system that is not properly accepted will not be used appropriately. As Van Der Laan et al. (1997) rightly point out, “it is unproductive to invest effort in designing and building an intelligent co-driver if the system is never switched on, or even disabled.” This observation has motivated the creation and validation of a scale designed to evaluate the level of acceptability of a system, with items assessing both satisfaction and usefulness (Van Der Laan et al., 1997). Finally, it should be noted that a poorly accepted system will generate problems related to the operator’s confidence in the machine’s capabilities, problems that may lead the operator to never delegate the task to the system. The link between sense of agency and acceptability was highlighted in a recent study showing that a diminished sense of agency in human–system interaction directly affects system acceptability (Le Goff et al., 2018). Overall, the lack of acceptability affects the quality of interactions between human operators and automated systems. In this article, we focus on the link between the lack of acceptability of systems and the incomplete, sometimes abnormal, experience of agency among operators interacting with these systems. We propose that it is possible to reduce lack of acceptability by restoring operators’ sense of agency via the communication of explanations related to how system decisions are made (see Section “How to improve human–Artificial Intelligence interactions?”). We suggest that the sense of agency is the latent psychological variable that mediates the link between system explicability and system acceptability (Figure 1).

- *Experience of agency and operator involvement in the task:* Any change in self-agency can modulate the operator’s involvement in the task at hand. In particular, human–machine interface research has shown that driving support effectively decreases driver control activity (Mulder et al., 2012) and that this decrease is linked to driver disengagement (Navarro et al., 2016). As an illustration, a recent study showed that even when vehicle supervision successfully reminded drivers to hold the wheel and look at the road, people still did not engage in driving and were unable to prevent the vehicle from crashing into a conflicting object (Victor et al., 2018). Yet, it is known that a reliable sense of agency is essential for the attribution of causal, but also moral and legal, responsibility (Bigenwald and Chambon, 2019). Furthermore, a relationship between sense of agency, motivation, and willingness to exert effort has been demonstrated (Eitam et al., 2013; Wang et al., 2019). A loss of agency could therefore constitute a form of moral disengagement from our actions that would disturb the mechanism classically used to regulate human behavior (Bandura, 1999). This disengagement can be problematic when operator decision-making is required (Wen et al., 2019) and leads to difficulties when the operator must regain manual control (Navarro et al., 2016). Notably, a previous study on emergency braking when using cruise control found that reaction time for braking was significantly longer when people used cruise control rather than manually controlling vehicle speed (Jammes et al., 2017).

- *Experience of agency and operational performance*: A change in the feeling of agency could have a direct influence on cognition, and through this, on operational performance. In particular, significant consequences in terms of attention and memory have been demonstrated. Thus, experimental work suggests that the sense of agency strongly influences attention allocation such that people effectively monitor events that are relevant to themselves (i.e., under their control), but do not pay much attention to events that are outside of their control (Wen and Haggard, 2018). Furthermore, studies investigating error-related potentials (i.e., brain activity associated with monitoring of the consequences of an action, e.g., San Martín, 2012, for a review) show a degradation of monitoring associated with a reduced sense of agency (Kühn et al., 2011; Li et al., 2011; Bednark and Franz, 2014; Timm et al., 2014; Caspar et al., 2016). Finally, Hon and Yeo (2021) report evidence that stimuli for which one feels a sense of agency are, in fact, remembered better than their counterparts without such a sense.

Thus, sense of agency may play an important role in the OOTL phenomenon, as well as in the level of acceptability of automated systems and the performance of the human operator. With the next generations of highly automated systems, a major challenge for the Human–Computer Interactions (HCI) community will consist in clarifying this relationship between automation and sense of agency. A better understanding of this interaction can provide a useful framework for thinking about interactions with automated technology and, in particular, for optimizing human–automation interactions.

### Sense of agency: What is it, and how does it develop?

As mentioned above, the sense of agency is traditionally defined as the experience of controlling one’s actions, and, through them, events in the external world (Haggard and Chambon, 2012). A functional sense of agency allows individuals to distinguish events that they have caused from those for which they are not responsible, but which, for example, were caused by chance of by other agents. As such, a sense of agency is considered a cornerstone of human experience (van Hateren, 2015; Haggard, 2017) and a deficient sense of agency is associated with various disabling clinical conditions, such as depression (Haggard and Chambon, 2012) or schizophrenia (Voss et al., 2010; Garbarini et al., 2016). In today’s society – in which one’s actions can have consequences for the lives of others – causal attribution of behavior is essential. In particular, self-agency plays a central role in society as the basis for legal responsibility or fair retribution for the work done (Haggard, 2017; Hallett, 2018).

It has been proposed that the sense of agency is composed of various subcomponents such as sense of intentionality, sense of initiation, and sense of control (Pacherie, 2007). The sense of intentionality, first, would have three main sources: awareness of the goal of the action, awareness of the situated goal, and the basic sense of doing, which arises from a comparison between the predicted and actual states of the action (Pacherie, 2007). Second, the emergence of the sense of initiation for a movement would depend on the awareness of the predicted sensory consequences of the movement. Indeed, it has been shown that awareness of the initiation of a movement is reported by the agent between 80 and 200 ms before the movement actually occurs (Libet et al., 1983; Libet, 1985). Finally, although the sense of initiation is considered a crucial component of the sense of agency, it does not seem to offer the guarantee that the agent feels the author of the action. For example, an agent may sometimes have a sense of having initiated an action but have no effective control over its course. Conversely, if an unexpected event occurs, an agent may feel that they lose control of what happens (even if they initiated the action). This feeling can lead to a reduction, or even an abolition, of the individual’s sense of agency. Feeling in control throughout an action has been called “sense of control” (Pacherie, 2007).

We chose to approach the notion of sense of agency primarily from the perspective of sense of control. Indeed, insofar as we place ourselves in an interaction where a system makes decisions, the sense of initiation of the action – and, to a lesser extent, the sense of intentionality – is most often automatically attributed to the system. Our objective will not be to question the conditions of emergence of an “artificial” sense of intentionality or action initiation, but to examine the conditions necessary for the human operator to develop a feeling of control over the effects of an action taken by an automated system. This control can be illusory, i.e., it does not correspond to any objective control over the operation or task in progress. In human–system interaction, illusions of control are commonplace. Inducing such illusions raises obvious ethical questions, which must be weighed against the benefits that these illusions can provide, such as keeping the operator engaged in the control loop (Nakashima and Kumada, 2020). This engagement is essential to counteract the well-documented “out-of-the-loop” performance problem, where disengaged operators have difficulty detecting system errors or regaining control in emergency situations.”

#### Sense of agency develops along the intention-action-effect chain

**FIGURE 2:**
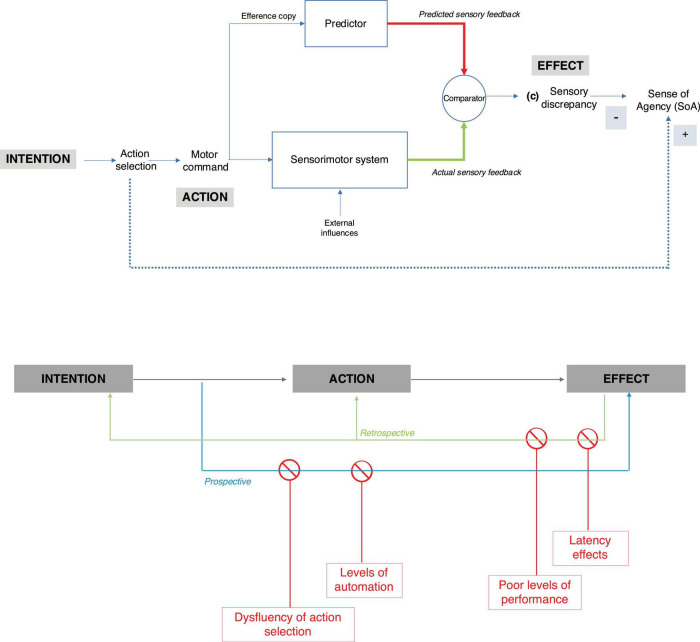
**(Top)** Computational model of the sense of agency (drawn using source data from Blakemore et al., 2002). **(Bottom)** Modulators of the sense of agency along the intention-action-effect chain (drawn using source data from Chambon and Haggard, 2012).

Normal human experience consists of a coherent flow of sensorimotor events, in which we first formulate action intentions and then move our bodies to produce a desired effect (Haggard et al., 2002). This involves linking our intentions to our actions, and our actions to their effects – i.e., the internal states they change or the events they produce in the external world. The chain “intention-action-effect” is thus key to developing a reliable sense of agency (Chambon and Haggard, 2013; Sidarus and Haggard, 2016). In particular, two dimensions of agentive experience have been highlighted: one prospective, the other retrospective (Figure 2).

Many studies have focused on the role of monitoring the consequences of our actions, i.e., on the *retrospective* dimension of sense of agency. Within this framework, it has been repeatedly shown that a sense of agency arises when external events following our action are consistent with predictions of action effects made by the motor system while we are performing or simply intending to perform an action (see Chambon et al., 2014a, for a review). It is also recognized that the neurocognitive mechanisms involved in the sense of agency rely on a variety of cues, i.e., internal cues such as motor signals or sensory feedback, and external cues such as situational cues or context (Moore and Fletcher, 2011). These cues help verify the integrity of the “intention-action-effect” causal chain, once the action is performed and its effects are known (that is, *retrospectively*). The brain’s agency system thus functions as a central processing system that brings together internal and external cues used in combination to establish the most robust agency representation (Moore et al., 2009). Multiple cues contribute to the sense of agency and cue integration approaches have been shown to be effective in modeling both human perception and action experience (Moore and Fletcher, 2011).

Another key dimension of agency has been emphasized more recently: the *prospective* dimension, which focuses on the intention-action link in the “intention-action-effect” chain mentioned above. This dimension of agentive experience concerns the processes that take place prior to action, and thus prior to receiving information about the actual effect of the action (Wenke et al., 2010; Chambon and Haggard, 2012). According to the prospective view of the sense of agency, the ease (or difficulty) with which an action is prepared, or selected from several possible actions, could also boost (or reduce) the sense of agency. This suggests that the sense of agency does not only depend on retrospective signals comparing predicted and actual sensory feedback, but also emerges from internal circuits related to action preparation and selection. In other words, the sense of agency could also rely on an early signal generated while we prepare or select the appropriate action to take.

Importantly, the sense of agency has been shown to be a flexible mechanism that can be modulated under certain conditions. In the next section, we examine different modulators of the sense of agency and discuss how automation of all or part of the intention-action-effect causal chain is likely to interact with them.

#### Degree of involvement and level of automation

It has been shown that user involvement in a task is critical for their perceived control. Indeed, a greater involvement of the user in an action increases the perceived control of this action (Baronas and Louis, 1988). Engagement in a task can be modulated by varying the intentionality of the action itself. For example, Caspar et al. (2018) showed a reduced sense of agency in agents receiving coercive instructions to perform an action compared to situations where they freely chose the action to perform. They also found a reduced sense of agency in commanders who coerced agents to perform an action compared to situations where they acted on their own. Ordering someone to do something thus decreases the individual sense of agency for that action and thus inevitably reduces the sense of responsibility for that action (Caspar et al., 2018). This link between sense of agency and freedom of action choice was also highlighted by Barlas and Obhi (2013) in an experiment varying the number of alternatives. They recorded a stronger sense of agency among participants when the number of alternatives was maximal, an intermediate sense of agency when participants had a medium level of action choices, and a weaker sense of agency when they had only one choice available to them. This result suggests that reduced opportunities for voluntary action selection, and thus indirectly a lower degree of investment in action, decreases the sense of agency.

In human–machine interactions, the degree of involvement in the task varies according to the degree of automation implemented. A number of studies suggest that increasing the level of automation proportionally decreases the development of the operator’s sense of agency. For example, Berberian et al. (2012) studied participants’ sense of agency while performing an aircraft supervision task using a flight simulator under different levels of automation. The task required the participant to observe a flight plan and after a random time interval, a conflict occurred due to the presence of another aircraft. The participant then had to decide on an appropriate command and execute it using a button interface. Following an established classification (Sheridan and Verplank, 1978), the task included different levels of automation, from the user having full control (no automation) to the computer performing the entire task with the participant merely observing (full automation). The authors found a decrease in self-agency concomitant with increasing level of automation, and argued that the increasing level of automation tended to distract operators from the results of their action, decreasing their sense of agency and thus disrupting their overall performance.

In a similar vein, Yun et al. (2018) measured drivers’ sense of agency during assisted and automated driving in a driving simulator. Their results showed that in the assisted driving condition, the sense of agency was significantly lower than in the manual driving condition (Yun et al., 2018). In addition, Mulder et al. (2012) showed that driving assistance effectively decreased driver control activity (Mulder et al., 2012) and was related to driver disengagement (Navarro et al., 2016).

Together, these results suggest that it is not the level of automation itself that modulates the sense of control in the first place, but rather the amount of control remaining over the action. One possible hypothesis to explain this decrease in the sense of control when interacting with automated systems is that the amount of control remaining is inversely proportional to the level of assistance (Berberian et al., 2012). On the one hand, in situations where the level of automation is low and when actions are still performed by a human agent, the agent’s sense of agency is not or minimally altered. On the other hand, in situations where automation is predominant (e.g., a supervisory task), the agent’s decision has less weight or is even useless, preventing the agent from developing control over the action performed. In this case, the development of a sense of agency is hindered by the high degree of automation.

#### Time course of voluntary actions and latency effects

The time course of the intention-action-effect chain is a key variable in the development of a sense of agency in human agents. Thus, increasing the interval between action and effect (i.e., delay) is a procedure often used to weaken the sense of agency in laboratory experiments. This manipulation of the sense of agency is derived from a classic self-touch delay paradigm, although the original study did not directly examine the sense of agency. Blakemore et al. (1999) have shown experimentally that increasing the delay between a self-produced action and the resulting tactile stimulus decreases the attribution of the action to the self (Blakemore et al., 1999). It has been argued that increasing the delay increases the intensity of the sensation because the stimulus no longer matches (in time) the motor command. Since then, many studies have shown a gradual decrease in the sense of agency as delay increases (Sato and Yasuda, 2005; Ebert and Wegner, 2010; Kühn et al., 2011; Farrer et al., 2013; Hon et al., 2013; Kawabe, 2013; Wen, 2019).

While most studies have focused on the impact of the delay between action and its consequences, some studies have also highlighted the role of the delay between intention and action on the feeling of agency. This is particularly true of Wegner’s work on the priority principle (Wegner and Wheatley, 1999). The priority principle requires that the perceived causes that precede an action be temporally closely related to the consequences of that action. The intention to act must have been formed just before the action is performed. In other words, for an agent to feel control over an action, the intention that initiates that action must occur within a sufficiently short period of time before the actual execution of the action (Hindriks et al., 2011).

Interestingly, the temporality of action is also considered a major issue in the field of ergonomics. Better known as latency – the time that elapses between the moment a device is physically moved and the moment the corresponding update appears on the screen (Foxlin, 2002) –, the delay between action and its consequences is a critical factor for the quality of HCI and a major bottleneck for usability (MacKenzie and Ware, 1993). Although a number of studies have shown that, in HCI situations, a longer interval between action and effect is associated with a lower sense of agency (Berberian et al., 2013; Wen et al., 2015b), delay influences the measures of agency via multiple possible processes, such as graded response, task performance, sensory pre-activation, and temporal perceptual sensitivity (see Wen (2019) for a review). The presence of a delay between the action and its consequence remains, however, essential to account for alterations of the agentive experience due to the increased latencies introduced by automation in the intention-action-effect chain.

Finally, while time is an essential component in the development of a sense of agency, it can be noted that being the intentional agent of an action can also modulate our temporal perception of the events surrounding our actions. Specifically, in the case of a voluntary (vs. involuntary) action, the perceived onset of the action is shifted toward the perceived onset of its consequence, and vice versa, resulting in a “compression” of the perceived temporal delay between the action and its outcome (Haggard et al., 2002; Di Costa et al., 2018). This subjective compression is referred to as the “intentional binding” phenomenon (Haggard et al., 2002). In addition to explicit (self-reported) measures (Wenke et al., 2010; Sidarus et al., 2017; Barlas and Kopp, 2018), intentional binding is now extensively used as an implicit measure of the sense of agency (see Moore and Obhi, 2012, for a review).

#### Performance levels and automation

Metcalfe and Greene (2007) were the first to show that agency judgments are strongly correlated with performance on a task, even when participants know that their performance is largely due to external factors (Metcalfe and Greene, 2007). More recently, van der Wel et al. (2012) also showed a significant negative correlation between participants’ error scores and their agency ratings. These results highlight another component of Wegner’s theory of apparent mental causation (Wegner and Wheatley, 1999): the consistency principle. As participants perform better on the task, their performance expectations likely become more consistent with actual performance, resulting in a greater sense of agency (van der Wel et al., 2012).

This relationship between performance and agency experience is also present in our interactions with machines. Wen et al. (2015a) first showed that level of performance produced by an action might be more important than action–outcome association in modulating the operator’s sense of agency. Indeed, if automation removes some control from users, it also provides more reliable and secure control over outcomes. The authors observed that participants’ sense of agency increased with better performance in an assisted condition compared to a self-control condition, even though a large portion of their commands were not executed (Wen et al., 2015a). In a goal-directed motor task in which computer assistance ignored erroneous user commands, Wen et al. (2015a) also showed that both sense of agency and performance could be improved by automation. In this task, computer assistance thus significantly improved performance and sense of agency compared to the condition in which all user commands were executed (Wen et al., 2015a; Endo et al., 2020).

With a similar experiment as Wen et al. (2015a); Inoue et al. (2017) showed that the sense of agency increases when performance improves even if participants were explicitly given the instruction about the presence of the assistance before the experimental task. Interestingly, the increase in sense of agency was maintained even when participants were told that their improved performance was due to the assistance of the automated system. These results suggest that even when there is a plausible cause of performance improvement other than one’s own action, the improvement may be misattributed to one’s own control, resulting in an increased sense of agency on the part of the human operator (Inoue et al., 2017).

In a similar vein, Ueda et al. (2021) showed that human operators’ sense of agency could be enhanced while maximum performance improvement is produced by an automated system. By implementing a tracking task in which participants had to continuously track a moving target via a joystick-controlled cursor under different levels of automation, the authors showed that participants’ sense of agency and tracking performance were improved as a function of automation level. Specifically, these results suggest that allowing operators to contribute slightly to monitoring the ongoing operation of an automated tool while achieving maximum performance improvement may be an effective solution to maintaining their sense of agency (Ueda et al., 2021).

Consistent with these results, Tanimoto et al. (2017) showed that a semi-automatic system that combined an ideal work trajectory with the operator’s manual trajectory maintained the operator’s sense of agency at a high level, similar to that of manual control, while improving performance at the same time (Tanimoto et al., 2017). More importantly, Tanimoto et al. (2017) also found that the sense of agency was strongly weakened if the semi-automatic system performed goal-directed assistance (providing a distal outcome) rather than trajectory assistance (providing a proximal outcome). This result is consistent with findings from experimental psychology that both proximal and distal action outcomes are important for developing a reliable sense of agency (Metcalfe et al., 2013; Vinding et al., 2013).

In summary, a system that improves the performance of a human operator also increases the operator’s sense of agency. More interestingly, studies show that the operator’s sense of agency is enhanced even in situations where improving performance involves ignoring some of the operator’s behavior – and thus ultimately reducing the actual control the operator has over the action.

#### Action-choice facilitation and readability of automated system choices

Experimental studies have shown that predictability influences the sense of agency. By manipulating the congruence between subliminal primes and the selection of a motor response on a keyboard, Wenke et al. (2010) showed that compatible primes did not facilitated responding by speeding up response times, but were associated with a higher sense of control (Wenke et al., 2010). Chambon and Haggard (2012) suggested that this priming effect was independent of motor execution itself but stemmed from the ease of action selection induced by the prime-target compatibility (Chambon and Haggard, 2012; see also Chambon et al., 2013; Chambon et al., 2015). Building on this work on action selection and self-agency, Sidarus et al. (2013) designed a task in which both the facilitation of action selection (compatible or incompatible primes) and the probability of occurrence of an effect, were varied. Importantly, they showed that an expected effect led to a higher evaluation of the feeling of control than an unexpected effect. More interestingly, they observed an interaction effect between the facilitation of action selection and the probability of effect occurrence: when an action was followed by an expected effect, there was no difference in the evaluation of control whether the prime was congruent or incongruent. However, when the action produced an unexpected effect, congruent primes (i.e., action facilitation) resulted in a higher sense of control than incongruent ones (Sidarus et al., 2013).

While the ability to predict the outcome of our actions is central to the development of reliable experience of agency, it is also clear that advances in automation technology tend to develop automatic cascades and reaction chains that reduce or even eliminate predictability and result in unexpected events. Such opacity makes it difficult for the operator to relate the system intention to actual state and predict the sequence of events that will occur. This lack of transparency on how the system makes decisions, or simply operates, is considered a key factor in understanding the impact of automation on the operator’s sense of agency. The link between transparency of system intentions and the operator’s sense of control was highlighted in a recent study (Le Goff et al., 2018). Specifically, this study showed that providing informational messages containing a system’s intentions during a supervision task improved the acceptability of the automated system and the sense of control of the user supervising the system (Le Goff et al., 2018). Thus, displaying system intentions before an action is a good candidate for maximizing the experience of agency in supervision tasks, and for increasing system acceptability as well.

Taking together, these different results confirm that (1) the sense of agency is a flexible mechanism that can be modulated by multiple variables and (2) our interactions with technology can significantly alter the agentive experience. While these issues (i.e., level of automation, change in performance, latency, system opacity) are now relatively well documented, the evolution of technological systems generates new challenges for the human operator experience, which are directly related to the nature of our relationship with technology. Indeed, in all the examples we have mentioned, technology is perceived as a mere tool at the disposal of the human operator, rather than a full-fledged member of the interaction. With the advent of AI and the increasing autonomy of technological systems that accompanies it, artificial agent should no longer be seen only as servants but as a partner (McNeese et al., 2018). This development generates new interaction problems that may revive interest in the role of social context on the experience of control. Interestingly, several recent studies have shown that the presence of an interaction partner can alter the sense of agency. The following section aims to highlight the changes brought about by the advent of AI as a partner (rather than just a tool) in our relationship to artificial agents, and the potential impact of these changes on human agency.

## What is new with Artificial Intelligence?

Artificial Intelligence emerged in the 1950s and included in its initial definition elements related to learning, memory organization and reasoning (McCarthy et al., 1955). AI is now defined as a sub-discipline of computer science that aims to produce programs that simulate human intelligence (American Psychological Association, 2020, see (Box 1) for more details), i.e., to create systems capable of performing tasks that normally require human intelligence.

**Box 1. The variety of AI.**

The American Psychological Association identifies several areas in which AI plays a prominent role, including robotics, computer vision, machine learning, gaming and expert systems.

In these different fields, AI is sometimes equated with machine learning tools, and may or may not involve the use of unsupervised (bio-inspired) learning algorithms, such as artificial neural networks. Specifically, **Machine Learning** (ML) is defined as a computational process that “uses input data to achieve a desired task without being literally programmed (i.e., hard coded) to produce a particular outcome” (El Naqa and Murphy, 2015), while **Deep Learning** (DL) is the study of artificial neural networks and related machine learning algorithms that contain more than one hidden layer (Ongsulee, 2017).

In this typology, ML is a subcategory of AI, while DL is a special case of ML. The definition produced by this typology implies that both ML and DL algorithms are built from data, and they establish their own decision processes. In this article, we are interested in explanations that can reduce the opacity with which these processes operate. The problem of opacity is common to ML and DL (although it is compounded for DL networks, whose layer operations suffer from a known lack of interpretability). In the remainder of the article, we use the term “AI” to refer to any class of algorithm that suffers from such opacity in the processes that generate their output.

It is now commonplace to say that AI is becoming increasingly pervasive in our daily lives. Many of our actions are indeed mediated by decisions taken by AI and its use tends to be democratized in various fields such as transportation, security, medicine, finance, defense, etc. While the use of AI today may seem trivial, its use involves life-changing decisions for some people. AI technologies are in fact likely to make increasingly important decision in the coming decades, and it is therefore critical for ethical issues to consider the responsibility of actions arising from AI decisions. These actions still require the approval or at least the supervision of human operators, which raises the question of the new type of interaction with human operators that AI promotes.

The following sections aim at highlighting the singularity of AI compared to automated systems in general, and the new questions it raises for human–machine interactions.

### Artificial Intelligence: A new type of interaction with human agents

To better understand why the introduction of AI has initiated a new type of interaction with human agents, it is essential to revisit the difference between intelligent systems (such as AI) and automated systems. Bigenwald (2018) proposed a useful taxonomy to better capture the legal status of AI. This taxonomy defines three broad classes of artificial systems with their specific properties. First, *automated systems* are complex rule-based systems. Second, *autonomous systems* are systems capable of a certain degree of adaptability, learning and evolution, and are generally capable of goal-oriented behavior. Finally, *intelligent systems* are systems capable of performing human cognitive tasks, and for which the issue of opacity or the “black box” is problematic – i.e., some of the “reasoning” produced by intelligent programs is untraceable and/or confusing to the human mind (Bigenwald, 2018). If these distinctions and issues are considered at the legal level, they must also be considered when thinking about the interactions these systems are likely to have with human agents at the cognitive level.

Let us first note that the notion of determinism plays an important role in the differences between automation and AI. We mentioned that automated systems are capable of doing things automatically, but always following explicit decision rules. For these systems, the decision rules are clearly established and accessible, i.e., they are bound with explicit programming and rules, through which the information given as input to the system will produce a predictable – deterministic – output. In contrast, AI has the ability to use data to create not only its own ontologies, but also its own decision rules. Importantly, huge amounts of data are now available to AI equipped with new tools to process and make sense of it –neural networks, graphs or deep machine learning algorithms. As such, AI is not deterministic in that its decision rules are derived from the set of data used to train the system itself, and hence the system’s decisions will always involve a small amount of uncertainty – just as in the case of the human brain (Shekhar, 2019). Establishing its own decision rules has advantages for AI that have led to its widespread adoption. In particular, AI algorithms often perform better than deterministic algorithms that encounter problems of generalizability, adaptation to new contexts or learning transfer (Botvinick et al., 2019). This performance allows AI to be used in increasingly complex environments and situations. However, a consequence of this is that the inner workings of the AI and its decision-making processes very often remain inaccessible, if not difficult to represent in intelligible symbols. This is what is referred to as the opacity, or the “black box,” of AI. This opacity problem has been extensively discussed in the literature, in particular in relation to the performance rate of systems. Indeed, it has been shown by Gunning and Aha (2019) that a relationship exists between the performance level of an algorithm and its opacity.

In summary, AI systems are characterized by their non-deterministic, complex behavior and their autonomy. These characteristics translate into an increasing opacity of these systems for the human operator. At the same time, the increasing autonomy of artificial agents gives them a completely different role from the one initially assigned to the machine, so that artificial agents could now be considered more as partners than as simple tools. This results in the introduction of new coordination requirements and the emergence of new categories of problems due to failures in the human–AI relationship. It is therefore essential to consider the potential impact of the artificial agent, understood as a teammate, on the control experience. As such, studies on the development of agency during joint actions in a social context could shed new light on how human–AI interactions modify the sense of agency of human operators.

### From mediated-agency to social agency

#### Role of the social context on the sense of agency

In the last decade, the impact of a social context on the individual’s sense of agency – i.e., how sense of agency develops in our interactions with others – has gained considerable traction (see Brandi et al., 2019, for a review). An important finding is that the social context can diminish, or hinder, agentive experience. This finding has been popularized through the notion of “diffusion of responsibility” – the idea that the presence of others modifies the individual’s behavior by making them feel less responsible for the consequences of their actions (Bandura et al., 1996). Similarly, acting under coercion has recently been shown to alter the subjective experience of being the author of an action. Thus, Caspar et al. (2016) showed that the neural processing of the outcome of an action performed under coercion was more similar to that observed during a passive movement than during an intentional action (Caspar et al., 2016; see also Sidarus et al., 2019, for an effect of “forced choice” on learning). In addition, the presence of others would lead agents to feel less responsible for the outcome of group decisions, especially those with negative consequences (Mynatt and Sherman, 1975; Forsyth et al., 2002). The presence of others also reduces agency by increasing the ambiguity of authorship, i.e., by weakening the neural linkage between one’s actions and their outcomes (Beyer et al., 2017). Finally, the presence of another individual (Beyer et al., 2017) or a robot (Ciardo et al., 2018) has been shown to decrease the sense of agency over external events, even when people actually have full control. In short, under ordinary circumstances, the sense of agency is generally lower when people share control with robots, compared to the condition in which people perform actions alone. It should be noted that the opposite effect has also been demonstrated in the experimental literature. Wegner et al. (2004) first demonstrated that the existence of a “vicarious” agency in a social context. In a seminal experiment, they showed that the sense of agency was increased when participants heard some instructions for an action, even if the action was performed by another person hidden from their view. This result is one of the first to suggest that we can develop a sense of agency for an action performed by a third party (Wegner et al., 2004).

If the presence of other people (human or artificial) modulates individuals’ sense of agency, our interactions with others also have a significant role in how our experience of control develops. Some daily tasks involve coordinating our efforts with others to achieve a common goal: “joint action” has been defined as any form of social interaction whereby two or more individuals coordinate their actions in space and time to bring about a change in the environment (Sebanz et al., 2006). In other words, joint action refers to the interdependent interactions involving two (or more) co-agents whose actions are directed toward a common goal (Sahai et al., 2017).

A series of behavioral experiments has first shown that knowledge about a co-actor’s task affects the planning and performance of one’s own action, even when the other’s role need not to be considered (Sebanz et al., 2003, 2005; Atmaca et al., 2008). The results suggest that participants anticipate the other’s action, which increases their own tendency to act. Relatedly, Obhi and Hall (2011) showed that sense of agency could be enhanced by the actions of another person in a joint action task in which participants acted one after another. The results showed that both participants experienced a comparable sense of agency on the outcome produced. These findings support the idea that interaction partners process the causal link between each other’s actions and the sensory outcomes of those actions, which led the authors to support a “we-agency” hypothesis (Obhi and Hall, 2011). The existence of a “we-mode” in social interaction has been proposed to explain this modulation of the agentive experience in social context (Gallotti and Frith, 2013). Specifically, the “we-mode” is a mode during which people automatically track their co-actors’ attention (Samson et al., 2010; Böckler et al., 2012), their performance (Sebanz et al., 2005), and their beliefs (Apperly and Butterfill, 2009; Kovács et al., 2010).

Recently, Silver et al. (2020) have developed a theoretical framework that captures the different faces that the sense of agency can take in a social context. Specifically, the authors suggest that the wide variety of agentive experiences can be represented along a continuum, with cooperation as the primary dimension. In this continuum, the presence of cooperative elements within an interaction should enhance agency, whereas a social interaction with little or no cooperation should decrease agency. Because there is a great variety of possible responses from one agent to another, social contexts lead to highly unpredictable responses due to the reduced ability to make predictions about the other’s behavior. Silver et al.’s agentive continuum is as follows: the most embedded type of joint agency is the “**we-agency**.” In this particular instantiation of joint agency, the co-actors share a common agentive identity and a common goal, but the boundaries between self and other are also blurred. Note that here this blurring of the self is experienced positively (Wahn et al., 2018). Second, in interactions where the self/other distinction is intact (no ambiguity about the origin of the action), but where the agents are engaged in joint action, **shared agency** is induced: self-agency and joint agency coexist. **Vicarious agency** occurs when the result of another agent’s action is wrongly attributed to the self, while **violated agency** occurs when the result of our own action is wrongly attributed to another agent who did not directly cause the action. Finally, **interfered agency** occurs when goals are ill-defined, or when there is no cooperation, or when the actions of the other agent are unpredictable. In this case, the presence of another agent interferes with our own agency.

#### The construction of the “we-agency”

A critical question is that of the mechanisms underlying the emergence of this “we-agency” in joint actions. It has been proposed that the sense of agency in joint actions relies on the same principle of congruence between predicted and actual outcomes as that involved in individual actions (Pacherie, 2012). As previously mentioned, individual sense of agency develops along the intention-action-effect chain. Along this chain, the strength of an individual’s sense of agency for an action depends on how accurately their predictions about the consequences of their action (at the cognitive, perceptual, and sensorimotor levels) match its actual consequences.

While the same congruence principle likely applies to joint actions, prediction becomes a much more complex task in joint actions (Pacherie, 2014). Indeed, in joint actions, agents must not only predict the consequences of their own actions, but they must also make predictions for the actions of their coagents, and integrate the prediction of self and others to construct predictions about the co-action (Pacherie, 2012). Predictability of others’ actions has been shown to modulate joint agency (Bolt and Loehr, 2017). A higher sense of shared control was evidenced with a more predictable co-agent, showing that people rely on predictions of others’ actions to derive a sense of “we-agency” during interpersonal coordination. However, in joint actions, agents do not have direct access to the co-agent’s intentions, motor commands, or sensory feedback, making it impossible to accurately match sensory feedback to the co-agent’s action. In tasks involving joint actions, the attribution of control is usually vague as a result.

How does the prediction mechanism operate in joint action? People have a strong tendency to form shared task representations when co-acting – i.e., they take into account what those around them are doing or are expected to do. There is a level at which individual actions and those of co-actors are represented in a functionally equivalent way, as proposed by the “common coding” framework: humans can code the actions of others in terms of their goals, e.g., when they imitate their actions (Bekkering et al., 2000). Interestingly, goal sharing has been shown to improve motor accuracy and enhance the sense of agency for self-generated and observed actions, compared to a condition without goal sharing (Hayashida et al., 2021). A shared intention between co-actors (a decider and a follower) thus increases the sense of agency of each (van der Wel et al., 2012; van der Wel, 2015). Similarly, in a driving automation experiment, Wen et al. (2019) found that a driver’s sense of agency can be maintained at a high level if the automated co-actor shares the driver’s intention and the joint action achieves good performance (Wen et al., 2019).

Thus, the process of multiple integration put forward in the construction of the individual sense of agency would also be at work in our joint actions. However, while this integration mechanism is broadly similar, the nature of the information to which we have access and its reliability are radically different, so that the weighting of available cues is significantly different in joint and individual actions. Indeed, because people have access to perceptual, but not sensory reafferent, information about their partners’ actions, perceptual predictions likely play a more important role than sensorimotor predictions in the experience of agency in social context (Pacherie, 2012; van der Wel et al., 2012).

Finally, it should be noted that, while the social agency continuum mentioned above (Silver et al., 2020) precisely captures the different nuances of agentive experience in social interaction, it remains silent on the place of **mediated agency**. Mediated agency corresponds to actions performed by humans on the basis of a machine-mediated decision (Le Goff et al., 2015). If this category of mediated agency is not often addressed, it may be because machine–human interaction is often considered outside the sphere of social interactions.

**FIGURE 3:**
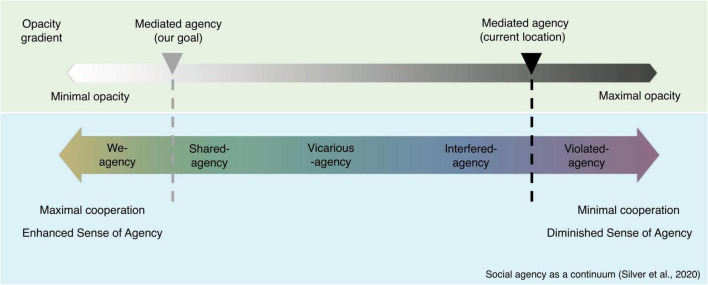
Location of the experience of mediated agency on the social agency continuum. Reproduced from Silver et al. (2020), with permission from Springer.

However, we believe that repositioning human–AI interactions on this continuum is relevant because of the characteristics endorsed by AI, such as autonomy and, to some extent, intentionality. It is likely that the location of “mediated agency” on the continuum is not fixed, but depends on the level of opacity of the system (Figure 3). Specifically, an opaque system may lead to shifting the experience of mediated agency to the right side of the continuum (e.g., “interfered agency”). Some of the methods and procedures described in what follows are specifically designed to communicate explanations that reduce the opacity of systems and, in so doing, reposition the sense of “mediated agency” to the left side of the continuum (the experience of “shared agency”).

## How to improve human–Artificial Intelligence interactions?

In this section, we discuss the field of XAI, a research area that attempts to make AI more readable and transparent to human agents by producing explanations on how AI makes decisions. We suggest that studying social interactions and understanding the development of human sense of agency in joint actions can help determine the content of explanations to be implemented in AI with the goal of improving human–AI interactions. In particular, since AI can be considered an intentional system, we suggest that providing access to different levels of intention (proximal and distal) implemented by AI could help restore human operators’ sense of agency, improve their confidence in the decisions made by artificial agents, and ultimately increase acceptability toward such agents.

### EXplainable Artificial Intelligence

The lack of transparency in many AI techniques discussed above has led to a growing interest in building explanations into AI systems to make their behavior interpretable and understandable (Putnam and Conati, 2019). What is now called XAI was first introduced in 1988 by Moore and Swartout, demonstrating a fairly early ambition for generating explanations related to the internal processes of a system (van Lent et al., 2004). Thus, an XAI is one that generates details or reasons to make its operation clear or easy to understand (Barredo Arrieta et al., 2019).

If the question of XAI is topical, we note that the reasons that motivate the explicability of artificial systems are mainly ethical or related to certification processes. The aim of “explanation” is therefore not to optimize the interaction between AI and its user but to give society confidence in the efficiency and rationality of the choices and behaviors resulting from these algorithms. On the other hand, few works have questioned the nature of the information to be provided to human operators to enable them to use these algorithms effectively, to understand them and to accept them.

In addition, providing information can also develop users’ understanding of the learning mechanism of an intelligent system (Putnam and Conati, 2019). The principle of “meaningful human control” over autonomous agents has been proposed (Santoni de Sio and van den Hoven, 2018). According to this principle, humans should ultimately remain in control of, and thus morally responsible for, algorithm decisions. This principle of “meaningful human control” is applicable provided that two conditions are met. First, a “tracking” condition whereby the system must be able to respond to both the relevant moral reasons of the humans designing and deploying the system and the relevant facts of the environment in which the system operates. Second, a “tracing” condition, according to which the system must be designed in such a way as to ensure that the outcome of its operations can always be traced back (Santoni de Sio and van den Hoven, 2018). Ultimately, we need to be able to push the responsibility for algorithmic outcomes back to humans, and algorithmic decisions must also follow human values. Robbins (2019) thus argues that meaningful human control is useful to enable humans to have the ability to accept, ignore, challenge, or override an AI algorithm’s decision.

Interestingly, among the different types of algorithms involving human–AI interactions, those aiming at recommending a choice to a user – i.e., recommendation systems – have started to integrate this notion of explanation in their algorithms. In this domain, explanations correspond to a description of the selected item that helps the user to “understand the qualities of the item well enough to decide whether or not it is relevant to them” (Tintarev and Masthoff, 2012). Some research has shown that presenting explanations to users can increase not only the persuasiveness of recommended items but also users’ trust and satisfaction with the recommendation system. Based on these results, guidelines have been developed for the design and evaluation of explanations for recommendation systems (Tintarev and Masthoff, 2010). In this respect, the line between explanation and manipulation is sometimes tenuous. The objective of explicability should not, however, be to present convincing-looking information that will lead operators to accept the proposals made by the algorithms without discussion. On the contrary, we advocate here to better identify and characterize what information is important for control, and what information the operator can rely on to understand the algorithm’s goals and how it wants to achieve them. It is precisely the role of explanation content that this article aims to highlight.

If the will to make AI systems more intelligible for humans is current, many uncertainties remain about how to make AI explainable. Indeed, aiming at making AI explainable and knowing how to do so are two very different issues. Miller (2018) was one of the first to make the connection between XAI and the social sciences, arguing that the field should build on existing research in this field, from philosophy to cognitive and social psychology. He also proposed that much of the work on XAI are based on researchers’ intuition about what constitutes a “good explanation,” i.e., one that disregards existing knowledge about individuals’ cognitive functioning (Miller, 2018; see also Bonnefon et al., 2016, for a call to rely on people’s moral intuitions in the field of driverless vehicles). Mueller et al. (2021), on the other hand, pointed out the lack of a scientific approach in the implementation of some XAI solutions. In particular, XAI systems are frequently algorithm-driven, that is, they start and end with an algorithm that implements a basic untested idea about explainability. The problem is that these systems are often not tested to determine whether the algorithm helps users achieve any goal, and so their explainability remains unproven (Mueller et al., 2021).

Finally, it should be noted that, since its emergence, the notion of XAI has often been invoked in work aimed at improving user confidence and satisfaction. However, XAI has paid little attention to an essential dimension of human cognition, namely the users’ sense of control. What would an XAI that promotes a sense of control in human operators look like? We believe that knowledge of the mechanisms underlying sense of control in joint action could shed new light on the nature of useful information to communicate to an AI user, and thus contribute to thinking about XAI, and autonomous artificial systems in general.

### Artificial Intelligence and intentionality

We recalled above that AI has a certain degree of decision-making autonomy. Interestingly, an autonomous technology can give the impression to the human operator that it has intentions. Whether or not machines can form an intention, i.e., an initial representation of a goal or state to be achieved, which precedes the initiation of the behavior itself (Pacherie, 2000), is open to debate. If we recognize that intentions are not of a single type but can be decomposed into different types and subtypes (depending on their complexity and temporal characteristics, see Pacherie, 2015; Chambon et al., 2017), then we can admit that some internal states of artificial systems satisfy some properties of low-level intentions, which they use to correct or adjust their actions or decisions when necessary. It is likely that it is because artificial systems have such internal representations that people make (sometimes delusional) inferences about those representations and attribute ‘intentional states’ to those systems.

The notion of “intentional stance” (Dennett, 1988) refers to a strategy of interpreting the behavior of an entity (person, animal, artifact) by treating it as if it were a rational agent that governs its action choices by taking into account its beliefs and desires (Dennett, 2009). An intrinsic disposition to attribute mental states (Kovács et al., 2010), combined with repeated exposure to intentional explanations during childhood, make humans experts at adopting the “intentional stance” when it comes to interpreting and predicting the behaviors of others (Perez-Osorio and Wykowska, 2020). Some experimental studies have shown that humans also adopt the intentional stance when interacting with robots, especially humanoids (Gazzola et al., 2007; Oberman et al., 2007). In particular, people may interpret robots in the same way as goal-directed agents (Wykowska et al., 2014), and they adopt an intentional stance toward the robot to a similar extent as they do when observing other humans (Thellman et al., 2017; Abubshait et al., 2021).

We argue here that, because of (i) the particular characteristics of AI in the landscape of artificial systems, and (ii) the frequent adoption of an intentional stance toward robots, the interactions that human operators have with AI share some properties with human social interactions. The intentional characteristics of AI thus lead us to consider human–AI interactions in the light of human–human relations. Since making the intentions of others clear and legible is important for human–human coordination, we will argue that doing the same with AI intentions is an essential condition for building reliable human–AI interactions.

### Improving human–Artificial Intelligence interactions: Displaying intentions along the goal hierarchy

We have seen that automation can significantly affect the sense of agency of human operators, not only because automation deprives the operator of the possibility of making choices themself, but also because the decisions made by automated systems – in particular autonomous and intentional systems – lack transparency, are inaccessible, or are not explicable at all (Norman et al., 1990). Such opacity makes it difficult for the operator to relate the system’s intention to actual state and to predict the sequence of events that will occur. Making the decisions of artificial systems more transparent, even “explaining” them, is therefore a crucial issue to restore the sense of agency of human operators. It is important to note that explanations are important not only for the quality of the interaction between operators and these systems – on which key dimensions such as operational performance and safety depend –, but also for the operators’ level of acceptability toward these systems. Here, we argue that the format and content of explanation could benefit from the study of social interactions in cognitive science.

#### The variety of intentions

As we have seen for joint actions, the sense of agency of the co-authors of a joint action increases when the co-authors share their intentions (Sebanz et al., 2003, 2005; Wegner et al., 2004; Atmaca et al., 2008; van der Wel et al., 2012; van der Wel, 2015, see also Le Bars et al., 2020). An obvious solution for the improvement of human–machine interactions would be to share the AI’s intentions with the operator. However, the question remains as to the modality of this sharing and, more critically, the format of the shared intention. Indeed, intention is not of a unique type but can be broken down into different types and subtypes. Crucially, some types of intentions might be easier to share than others.

Various models of goal-directed action, bringing together theoretical work on intentionality and empirical work on representations and motor control, have been proposed to account for the variety of intentions. One of the most influential distinguishes three classes of intentions – distal, proximal, and motor – according to the level they occupy in the hierarchy of action control (Pacherie, 2008; see Chambon et al., 2011, 2017, for empirical implementation). The central idea of this model, called “DPM model,” is that action control is the result of integrated and coordinated activity between these levels of intention (Mylopoulos and Pacherie, 2019). The DPM model specifies the representational and functional profiles of each type of intention as follows:

• Distal intentions (D-intentions) instantiate the most abstract level of representation of the action to be performed. D-intentions concern actions of endogenous origin that have a long-term, complex or abstract goal. Once formed, they also have the function of initiating a planning process – especially when the goal to be achieved is complex and novel – or, when dealing with goals for which one already has a plan of action, to retrieve this plan from memory (Khamassi and Pacherie, 2018). D-intentions can thus be both the result of a process of deliberation about ends (what to do?) and the starting point of processes of deliberation on means (how to do it?).
• Proximal intentions (P-intentions) instantiate finer and more immediate representations of the action to be performed. A proximal intention specifies the proposed action by anchoring it in the present situation and by selecting motor programs adapted to this situation. The representation of the action is further specified by the integration of perceptual information about the situation which will constitute as many constraints on the choice of the motor program to be implemented.
• Motor intentions (M-intentions) instantiate detailed sensorimotor representations of movements in space and the targets of those movements. M-intentions can be seen as being the action specification process, since they guide the action in real time: they encode action goals together with the motoric means for achieving them and do so in a motoric format directly suitable to action execution – meaning that only those attributes or properties of objects that are necessary for the specification of movements are encoded.

The meaning of “intention” clearly depends on which stages of the action specification process are being discussed. Some of the functions attributed to intentions are typically played in the period between the initial formation of the intention itself and the initiation of the action (D-intention level). In contrast, other functions—in particular, their role in guiding and monitoring the action—are played in the period between the initiation of the action and its completion (M-intention level). Communicating AI’s intentions at either of these levels can induce beneficial changes in the human operator’s self-agency. Thus, some studies have shown that successfully decoding operators’ motor intentions for manual action (via a Brain–Computer Interface) and transmitting then to the hand of humanoid robots induces in operators a sense of agency over the robot hand and an illusion of bodily ownership over it (Perez-Marcos et al., 2009; Slater et al., 2009; Alimardani et al., 2013). Interestingly, during robot embodiment, people generally do not feel that they are sharing control with the robot; instead, they feel that the robot’s hand is a part of their body and is under the full control of their own (Wen et al., 2019).

**FIGURE 4:**
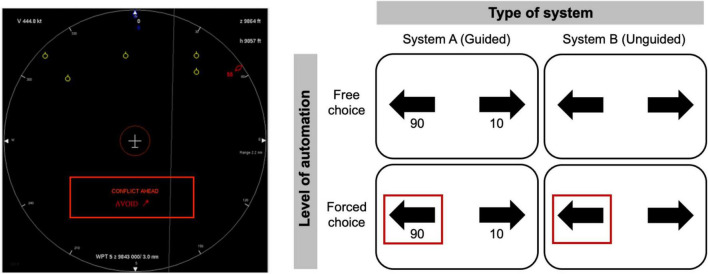
**(Left)** Illustration of a type of explanation used in Le Goff et al. (2018). The message ‘AVOID’ appeared together with an arrow, and a mark on the large white circle indicated the direction selected by the system. **(Right)** Illustration of a type of explanation used in Vantrepotte et al. (2022). Participants interacted with two different systems: a system (System A) guiding the subject by returning its relative confidence (between 0 and 100) in each of the two possible answers, and a system (System B) returning nothing (no guidance). Reproduced from in Le Goff et al. (2018), with permission from Taylor & Francis. Reproduced from Vantrepotte et al. (2022), with permission from Elsevier.

The previously mentioned study by Le Goff et al. (2018) aimed to directly investigate how the predictability of a system (Figure 4, left) – and specifically how messages conveying the system’s intent – can influence the development of agency in supervisory situations. The idea was to provide information about what the automated system is about to do next, and to observe whether this information improves user’s level of acceptability of the system as well as their sense of control. The results suggest that displaying the system’s intentions is an effective approach to improving users’ sense of control and acceptability toward the system. This study further shows that providing information about higher-order intentions (P-intentions), rather than just motor intentions (M-intentions, according to the DPM model), increases the sense of control of the action produced by an automated system, above and beyond improving bodily ownership. These works suggest that the presentation of the intentions of the system allows to reduce its opacity, in particular allows an anticipation of the actions planned by the system, allowing the implementation of the predictive mechanisms required for the retrospective dimension of sense of agency.

Importantly, the link between explanations and the experience of increased control suggests that the sense of agency is not only the product of a comparison between the predicted and achieved goal of an action, as the explanation itself is communicated before the action is performed or the decision is made. This prospective influence of the explanation on the sense of agency is reminiscent of the effect of action selection fluency on sense of agency, an effect that occurs before the action is performed before it is known whether or not the action goal has been achieved. Thus, it has been shown repeatedly that the sense of agency depends not only on a retrospective comparison mechanism between the predicted and achieved goal of the action, but also on prospective processes related to the experience of fluency, i.e., the ease or clarity with which an action is performed or a decision made (see Chambon et al., 2014a).

As mentioned earlier, to the extent that we interact with a system that makes decisions, the initiation of the action is most often automatically attributed to the system and, in this case, bodily ownership of the action is irrelevant. Human–machine interactions are particularly concerned with explicability at the most abstract (i.e., less concrete) levels of the action control hierarchy. Reflecting this concern for higher levels of action representation, Vantrepotte et al. (2022) recently explored the effect of communicating metacognitive information (Figure 4, right) on enhancing the feeling of control of participants interacting with a piloting assistance system. In particular, the authors investigated whether communicating the system’s confidence in its decision (rather than just the decision itself) could increase the system’s intelligibility and acceptability. The results showed that communicating the confidence – i.e., the degree of uncertainty – associated with the system choice not only enhanced the user’s sense of control, but also gave them greater confidence in the decision, and improved their performance (Vantrepotte et al., 2022).

Confidence can be seen as a measure of the uncertainty (or certainty) associated with one’s choice or action (Fleming and Lau, 2014). Communicating confidence was meant to improve explicability of the system’s decision by increasing its transparency, that is, by providing the participant with additional information about the decision itself (Tintarev and Masthoff, 2015). Indeed, the level of uncertainty (or confidence) associated with a decision is a key explanatory factor for why a decision is made or not, and whether or not that decision will be updated or revised in the future (Balsdon et al., 2020). The beneficial role of confidence on decision making has already been demonstrated in group settings, where the sharing of metacognitive representation increases joint performance (Bahrami et al., 2010; Fusaroli et al., 2012; Le Bars et al., 2020) and enhances team coordination (Lausic et al., 2009; Poizat et al., 2009; Le Bars et al., 2020). Communicating confidence also makes performance more fluid and prospectively improves the sense of agency (Chambon et al., 2014a,b) especially when sensorimotor information is not available (Pacherie, 2013), as when interacting with an automated system.

Interestingly, this method (communicating confidence) shares similarities with the counterfactual method proposed by Wachter et al. (2018). In the Vantrepotte’s study, the system communicates its confidence both in the action or choice finally selected by the operator (or realized by the system) but also in the unselected alternatives, i.e., the counterfactuals. Communicating information about both the final chosen option and the counterfactuals improves a number of key indicators (sense of control, acceptability, performance). In agreement with Wachter and colleagues, we believe that making unchosen alternatives explicit can help operators understand why a particular decision was made, and potentially provide reasons to challenge the decision if the outcome is not desired. Future work can test whether there is a number of alternatives for which these key indicators, instead of improving, deteriorate. Similar thoughts are being pursued in the area of causal inference, where it is shown that too many counterfactuals can alter the quality of causal inference, whereas they usually improve it (Lucas and Kemp, 2015).

#### Communicating the system’s intention to promote mind alignment

Gallotti et al. (2017) recently proposed that the nature of the information exchanged between interacting agents is crucial in determining the social or non-social nature of a human interaction, rather than the existence or absence of a shared goal. Their central claim is that social cognition is about the dynamic process of *aligning* individual minds, even in the absence of a shared goal. Such “mind alignment” emerges in social interactions involving the reciprocal exchange of information by which individuals adjust their minds and bodies in a gradual manner. To understand the nature of social interactions, the authors therefore propose to study how individuals align their words and thoughts, body postures and movements, in order to take into account the member(s) of the interaction and fully exploit socially relevant information (Gallotti et al., 2017).

Relatedly, we propose that the informational content to be conveyed to foster such mind alignment in HCIs is the intention of the AI system at either level of the intention hierarchy (distal, proximal, motor), depending on the need and specificity of the interaction at hand (whether to improve bodily ownership, task prediction, sense of control, and/or acceptability toward the artificial agent). A confidence measure associated with this intention, quantifying the extent to which the intention is likely to achieve the intended goal, would be a useful complement to the content of this communication. Future experiments should be conducted to test whether and how communicating intentions at the most abstract levels (D-intentions) of an AI system improves users’ sense of control over the effects of AI decisions, as well as the level of acceptability toward the artificial system.

Knowledge gained from cognitive science about how human operators develop a reliable sense of agency in social contexts can help decide what kind of explanation we want to provide in human–AI interactions. In particular, making AI intentions accessible at different levels of complexity can shift the cursor of experience in human–machine interactions from mediated agency to true joint agency (Silver et al., 2020). Doing so can make interactions with AI more social, and ultimately do justice to the novelty of AI’s status among artificial systems. Reconsidering the human–AI relationship as a particular type of social interaction, and thus AI as a partner to cooperate with rather than a tool, offers a valuable perspective for improving interactions between humans and advanced artificial systems, both present and future.

## Conclusion and perspectives

This article sheds new light on the study of human–AI interaction by arguing that the future of new technologies must be thought in the light of knowledge about human cognition, and in particular about the cognitive processes at play in social context. In particular, we argue that cognitive science insights into how human operators develop a reliable sense of agency in social contexts can help determine the kind of explanation we want to communicate in human–AI interactions.

First, we emphasized the importance of the sense of agency in the way human agents perceive and evaluate their own actions. We suggested that the sense of agency could be a good candidate to explain the difficulties observed in human–automated system interactions. We have shown that advances in automation technologies can disrupt the development of the experience of agency and that this disruption can have important operational consequences. Indeed, decrease in the sense of agency when interacting with highly automated systems has been shown to threat the acceptability of the system’s decisions by human operators. A diminished sense of agency can also lead to decreased involvement and motivation in the task at hand, as well as a decreased willingness to exert effort. A reduced sense of agency is known to have negative impact on attention and memory, and therefore on operational performance.

We then described how the sense of agency develops along the intention-action-effect causal chain in two complementary dimensions, one retrospective, the other prospective. We presented the sense of agency as a flexible mechanism that can be modulated under certain conditions, and discussed how the automation of all or part of the intention-action-effect causal chain is likely to interact with these conditions. We drew some parallels between the degree of operator involvement and the level of automation of a task, the importance of the temporal unfolding of voluntary actions and the potential latency effects induced by automation, the impact of performance on the sense of control and the increased levels of performance induced by automation, and finally between the facilitation of action choice and the readability of the choices of the automated system.

It is now clear that the evolution of technological systems generates new challenges for the human operator experience, which are directly related to the nature of our relationship with technology. We have therefore suggested that understanding the relationship between automation and the sense of agency is essential to optimizing human–automation interactions. Furthermore, with the advent of AI, technology will soon no longer be seen as a mere tool at the disposal of the human operator, but as an integral member of the interaction. We have thus highlighted the changes brought about by the advent of AI as a “partner” in our relationship with artificial agents, and the potential impact of these changes on human agency. We have detailed some of the existing differences between automated systems and AI. The characteristics of complexity, non-determinism and autonomy lead to an increasing opacity of these systems for the human operator, and ultimately give AI a completely different role from the one initially assigned to the machine. In particular, these characteristics have allowed us to raise the question of the readability of AI in a context of *joint action*.

Finally, we addressed the field of XAI through its goal of increasing the readability of AI algorithms by adding targeted explanations. An important contribution to this area could be to determine the content of explanations to be implemented in AI by understanding the development of the sense of human control in joint actions. What would an XAI that promotes a sense of control in human operators look like? We have shown that an autonomous technology has characteristics that encourage the human operator to view it as having intentions. The intentional characteristics of AI led us to consider human–AI interactions in the light of human–human relations. Since making the intentions of others clear and legible is important for human–human coordination, we argued that doing the same with AI intentions is an essential condition for building reliable human–AI interactions. We suggested that sharing AI intentions with the operator is a solution to consider for increasing the sense of control of human operators, focusing on a model that distinguishes different classes of intentions – distal, proximal, and motor – according to the level they occupy in the hierarchy of action control. The central idea of this model, called “DPM model,” is that action control is the result of an integrated and coordinated activity between these different levels of intention. Future work could systematically test the effect of communicating system intent at any of these levels, both in terms of benefits to the human operator’s agency but also to the operator’s levels of acceptability and confidence in the AI’s decisions.

## Author contributions

MP wrote the first draft of the manuscript. VC and BB reviewed the manuscript. All authors have read and approved the submitted version.

## References

[B1] Abubshait A. Pérez-Osorio J. De Tommaso D. Wykowska A. (2021). “Collaboratively framed interactions increase the adoption of intentional stance towards robots,” in *Proceedings of the 2021 30th IEEE international conference on robot & human interactive communication (RO-MAN)*, (Vancouver, BC), 10.31219/osf.io/zwqfa

[B2] Alimardani M. Nishio S. Ishiguro H. (2013). Humanlike robot hands controlled by brain activity arouse illusion of ownership in operators. *Sci. Rep.* 3:2396. 10.1038/srep02396 23928891 PMC3738936

[B3] American Psychological Association (2020). *Artificial intelligence.* Worcester, MA: American Psychological Association.

[B4] Apperly I. A. Butterfill S. A. (2009). Do humans have two systems to track beliefs and belief-like states? *Psychol. Rev.* 116 953–970. 10.1037/a0016923 19839692

[B5] Atmaca S. Sebanz N. Prinz W. Knoblich G. (2008). Action co-representation: The joint SNARC effect. *Social Neuroscience* 3 410–420. 10.1080/17470910801900908 18633833

[B6] Bahrami B. Olsen K. Latham P. E. Roepstorff A. Rees G. Frith C. D. (2010). Optimally interacting minds. *Science* 329 1081–1085. 10.1126/science.1185718 20798320 PMC3371582

[B7] Balsdon T. Wyart V. Mamassian P. (2020). Confidence controls perceptual evidence accumulation. *Nat. Commun.* 11:1753. 10.1038/s41467-020-15561-w 32273500 PMC7145794

[B8] Bandura A. (1999). *Handbook of personality, second edition: theory and research.* Amsterdam: Elsevier.

[B9] Bandura A. Barbaranelli C. Caprara G. V. Pastorelli C. (1996). Mechanisms of moral disengagement in the exercise of moral agency. *J. Personal. Soc. Psychol.* 71 364–374.

[B10] Barlas Z. Kopp S. (2018). Action choice and outcome congruency independently affect intentional binding and feeling of control judgments. *Front. Hum. Neurosci.* 12:137. 10.3389/fnhum.2018.00137 29695958 PMC5904194

[B11] Barlas Z. Obhi S. S. (2013). Freedom, choice, and the sense of agency. *Front. Hum. Neurosci.* 7:514. 10.3389/fnhum.2013.00514 24009575 PMC3756740

[B12] Baronas A.-M. K. Louis M. R. (1988). Restoring a sense of control during implementation: How user involvement leads to system acceptance. *MIS Q.* 12 111–124. 10.2307/248811

[B13] Barredo Arrieta A. Díaz-Rodríguez N. Del Ser J. Bennetot A. Tabik S. Barbado A. (2019). Explainable artificial intelligence (XAI): Concepts, taxonomies, opportunities and challenges toward responsible AI. *arXiv* [Preprint] 10.48550/arXiv.1910.10045 35895330

[B14] Bednark J. G. Franz E. A. (2014). Agency attribution: Event-related potentials and outcome monitoring. *Exp. Brain Res.* 232 1117–1126. 10.1007/s00221-014-3821-4 24504195

[B15] Bekkering H. Wohlschlager A. Gattis M. (2000). Imitation of gestures in children is goal-directed. *Q. J. Exp. Psychol. Sect. A* 53 153–164. 10.1080/713755872 10718068

[B16] Berberian B. (2019). Man-machine teaming: A problem of agency. *IFAC-PapersOnLine* 51 118–123. 10.1016/j.ifacol.2019.01.049

[B17] Berberian B. Le Blaye P. Schulte C. Kinani N. Sim P. R. (2013). “Data transmission latency and sense of control,” in *Engineering psychology and cognitive ergonomics. understanding human cognition*, Vol. 8019 ed. Harris D. (Berlin: Springer), 3–12. 10.1007/978-3-642-39360-0_1

[B18] Berberian B. Sarrazin J.-C. Le Blaye P. Haggard P. (2012). Automation technology and sense of control: A window on human agency. *PLoS One* 7:e34075. 10.1371/journal.pone.0034075 22479528 PMC3316600

[B19] Beyer F. Sidarus N. Bonicalzi S. Haggard P. (2017). Beyond self-serving bias: Diffusion of responsibility reduces sense of agency and outcome monitoring. *Soc. Cogn. Affect. Neurosci.* 12 138–145. 10.1093/scan/nsw160 27803288 PMC5390744

[B20] Bigenwald A. (2018). “The legal challenge of civil liability in the age of artificial intelligence: the autonomous robot?: Person, slave or machine?,” in *Proceedings of the international association for computing and philosophy*, Warsaw.

[B21] Bigenwald A. Chambon V. (2019). Criminal responsibility and neuroscience: No revolution yet. *Front. Psychol.* 10:1406. 10.3389/fpsyg.2019.01406 31316418 PMC6610327

[B22] Blakemore S.-J. Frith C. D. Wolpert D. M. (1999). Spatio-temporal prediction modulates the perception of self-produced stimuli. *J. Cogn. Neurosci.* 11 551–559. 10.1162/089892999563607 10511643

[B23] Blakemore S.-J. Wolpert D. M. Frith C. D. (2002). Abnormalities in the awareness of action. *Trends Cogn. Sci.* 6, 237–242. 10.1016/S1364-6613(02)01907-1 12039604

[B24] Böckler A. Knoblich G. Sebanz N. (2012). Effects of a coactor’s focus of attention on task performance. *J. Exp. Psychol. Hum. Percept. Perform.* 38 1404–1415. 10.1037/a0027523 22409143

[B25] Bolt N. K. Loehr J. D. (2017). The predictability of a partner’s actions modulates the sense of joint agency. *Cognition* 161 60–65. 10.1016/j.cognition.2017.01.004 28110236

[B26] Bonnefon J.-F. Shariff A. Rahwan I. (2016). The social dilemma of autonomous vehicles. *Science* 352 1573–1576. 10.1126/science.aaf2654 27339987

[B27] Botvinick M. Ritter S. Wang J. X. Kurth-Nelson Z. Blundell C. Hassabis D. (2019). Reinforcement learning, fast and slow. *Trends Cogn. Sci.* 23 408–422. 10.1016/j.tics.2019.02.006 31003893

[B28] Brandi M.-L. Kaifel D. Bolis D. Schilbach L. (2019). The Interactive self – a review on simulating social interactions to understand the mechanisms of social agency. *I-Com* 18 17–31. 10.1515/icom-2018-0018

[B29] Caspar E. A. Christensen J. F. Cleeremans A. Haggard P. (2016). Coercion changes the sense of agency in the human brain. *Curr. Biol.* 26 585–592. 10.1016/j.cub.2015.12.067 26898470 PMC4791480

[B30] Caspar E. A. Cleeremans A. Haggard P. (2018). Only giving orders? An experimental study of the sense of agency when giving or receiving commands. *PLoS One* 13:e0204027. 10.1371/journal.pone.0204027 30256827 PMC6157880

[B31] Chambon V. Domenech P. Jacquet P. O. Barbalat G. Bouton S. Pacherie E. (2017). Neural coding of prior expectations in hierarchical intention inference. *Sci. Rep.* 7:1278. 10.1038/s41598-017-01414-y 28455527 PMC5430911

[B32] Chambon V. Domenech P. Pacherie E. Koechlin E. Baraduc P. Farrer C. (2011). What are they up to? The role of sensory evidence and prior knowledge in action understanding. *PLoS One* 6:e17133. 10.1371/journal.pone.0017133 21364992 PMC3041795

[B37] Chambon V. Sidarus N. Haggard P. (2014a). From action intentions to action effects: How does the sense of agency come about? *Front. Hum. Neurosci.* 8:320. 10.3389/fnhum.2014.00320 24860486 PMC4030148

[B33] Chambon V. Filevitch E. Haggard P. (2014b). “What is the human sense of agency, and is it metacognitive?,” in *The cognitive neuroscience of metacognition*, eds Fleming S. Frith C. (Heidelberg: Springer), 10.1007/978-3-642-45190-4_14

[B34] Chambon V. Haggard P. (2012). Sense of control depends on fluency of action selection, not motor performance. *Cognition* 125 441–451. 10.1016/j.cognition.2012.07.011 22902284

[B35] Chambon V. Haggard P. (2013). “Chapter. 4 Premotor or ideomotor: how does the experience of action come about?,” in *Action science foundation of an emerging discipline*, eds Prinz W. Beisert M. Herwig A. (Cambridge, MA: MIT Press), 359–380. 10.7551/mitpress/9780262018555.001.0001

[B36] Chambon V. Moore J. W. Haggard P. (2015). TMS stimulation over the inferior parietal cortex disrupts prospective sense of agency. *Brain Struct. Funct.* 220 3627–3639. 10.1007/s00429-014-0878-6 25134684

[B38] Chambon V. Wenke D. Fleming S. M. Prinz W. Haggard P. (2013). An online neural substrate for sense of agency. *Cerebral Cortex* 23 1031–1037. 10.1093/cercor/bhs059 22510529

[B39] Christoffersen K. Woods D. (2002). “How to make automated systems team players,” in *Advances in human performance and cognitive engineering research*, Vol. 2 ed. Stone D. (Amsterdam: Elsevier), 1–12. 10.1016/S1479-3601(02)02003-9

[B40] Ciardo F. De Tommaso D. Beyer F. Wykowska A. (2018). “Reduced sense of agency in human-robot interaction,” in *Social robotics*, eds Ge S. S. Cabibihan J.-J. Salichs M. A. Broadbent E. He H. Wagner A. R. (Cham: Springer International Publishing), 441–450. 10.1007/978-3-030-05204-1_43

[B41] Dekker S. W. A. Woods D. D. (2002). MABA-MABA or abracadabra? Progress on human-automation co-ordination. *Cogn. Technol. Work* 4 240–244. 10.1007/s101110200022

[B42] Dennett. (1988). Précis of the intentional stance. *Behav. Brain Sci*. 11:495. 10.1017/S0140525X00058611

[B43] Dennett D. (2009). “Intentional systems theory,” in *The oxford handbook of philosophy of mind*, ed. Beckermann A. (Oxford: Oxford University Press), 10.1093/oxfordhb/9780199262618.003.0020

[B44] Di Costa S. Théro H. Chambon V. Haggard P. (2018). Try and try again: Post-error boost of an implicit measure of agency. *Q. J. Exp. Psychol.* 71 1584–1595. 10.1080/17470218.2017.1350871 28697690

[B45] Ebert J. P. Wegner D. M. (2010). Time warp: Authorship shapes the perceived timing of actions and events. *Conscious. Cogn.* 19 481–489. 10.1016/j.concog.2009.10.002 19896868 PMC2836403

[B46] Eitam B. Kennedy P. M. Tory Higgins E. (2013). Motivation from control. *Exp. Brain Res.* 229 475–484. 10.1007/s00221-012-3370-7 23288323

[B47] El Naqa I. Murphy M. J. (2015). “What is machine learning?,” in *Machine learning in radiation oncology: theory and applications*, eds El Naqa I. Li R. Murphy M. J. (Cham: Springer International Publishing), 3–11. 10.1007/978-3-319-18305-3_1

[B48] Endo S. Fröhner J. Musić S. Hirche S. Beckerle P. (2020). Effect of external force on agency in physical human-machine interaction. *Front. Hum. Neurosci.* 14:114. 10.3389/fnhum.2020.00114 32457587 PMC7227379

[B49] Endsley M. R. (1999). Level of automation effects on performance, situation awareness and workload in a dynamic control task. *Ergonomics* 42 462–492. 10.1080/001401399185595 10048306

[B50] Endsley M. R. Kiris E. O. (1995). The out-of-the-loop performance problem and level of control in automation. *Hum. Fact. J. Hum. Fact. Ergono. Soc.* 37 381–394. 10.1518/001872095779064555

[B51] Ephrath A. R. Young L. R. (1981). “Monitoring vs. man-in-the-loop detection of aircraft control failures,” in *Human detection and diagnosis of system failures*, eds Rasmussen J. Rouse W. B. (Springer: Boston, MA), 143–154. 10.1007/978-1-4615-9230-3_10

[B52] Farrer C. Valentin G. Hupé J. M. (2013). The time windows of the sense of agency. *Conscious. Cogn.* 22 1431–1441. 10.1016/j.concog.2013.09.010 24161792

[B53] Fleming S. M. Lau H. C. (2014). How to measure metacognition. *Front. Hum. Neurosci*. 8:443. 10.3389/fnhum.2014.00443 25076880 PMC4097944

[B54] Forsyth D. R. Zyzniewski L. E. Giammanco C. A. (2002). Responsibility diffusion in cooperative collectives. *Personal. Soc. Psychol. Bull.* 28 54–65. 10.1177/0146167202281005

[B55] Foxlin E. (2002). “Motion tracking requirements and technologies,” in *Handbook of virtual environments*, ed. Stanney K. (Boca Raton, FL: CRC Press).

[B56] Fusaroli R. Bahrami B. Olsen K. Roepstorff A. Rees G. Frith C. (2012). Coming to terms: quantifying the benefits of linguistic coordination. *Psychol. Sci.* 23 931–939. 10.1177/0956797612436816 22810169

[B57] Gallotti M. Fairhurst M. T. Frith C. D. (2017). Alignment in social interactions. *Conscious. Cogn.* 48 253–261. 10.1016/j.concog.2016.12.002 28033550

[B58] Gallotti M. Frith C. D. (2013). Social cognition in the we-mode. *Trends Cogn. Sci.* 17 160–165. 10.1016/j.tics.2013.02.002 23499335

[B59] Garbarini F. Mastropasqua A. Sigaudo M. Rabuffetti M. Piedimonte A. Pia L. (2016). abnormal sense of agency in patients with schizophrenia: Evidence from bimanual coupling paradigm. *Front. Behav. Neurosci.* 10:43. 10.3389/fnbeh.2016.00043 27014005 PMC4783405

[B60] Gazzola V. Rizzolatti G. Wicker B. Keysers C. (2007). The anthropomorphic brain: The mirror neuron system responds to human and robotic actions. *NeuroImage* 35 1674–1684. 10.1016/j.neuroimage.2007.02.003 17395490

[B61] Grynszpan O. Sahaï A. Hamidi N. Pacherie E. Berberian B. Roche L. (2019). The sense of agency in human-human vs human-robot joint action. *Conscious. Cogn.* 75:102820. 10.1016/j.concog.2019.102820 31561189

[B62] Gunning D. Aha D. (2019). DARPA’s explainable artificial intelligence (XAI) program. *AI Magazine* 40 44–58. 10.1609/aimag.v40i2.2850

[B63] Haggard P. (2017). Sense of agency in the human brain. *Nat. Rev. Neurosci.* 18 196–207. 10.1038/nrn.2017.14 28251993

[B64] Haggard P. Chambon V. (2012). Sense of agency. *Curr. Biol.* 22 R390–R392. 10.1016/j.cub.2012.02.040 22625851

[B65] Haggard P. Clark S. Kalogeras J. (2002). Voluntary action and conscious awareness. *Nat. Neurosci.* 5 382–385. 10.1038/nn827 11896397

[B66] Hallett N. (2018). Psychiatric evidence in diminished responsibility. *J. Crim. Law* 82 442–456. 10.1177/0022018318801677

[B67] Hayashida K. Nishi Y. Osumi M. Nobusako S. Morioka S. (2021). Goal sharing with others modulates the sense of agency and motor accuracy in social contexts. *PLoS One* 16:e0246561. 10.1371/journal.pone.0246561 33539426 PMC7861436

[B68] Hindriks K. Wiggers P. Jonker C. Haselager W. (2011). “Towards a computational model of the self-attribution of agency,” in *Modern approaches in applied intelligence*, eds Mehrotra K. G. Mohan C. K. Oh J. C. Varshney P. K. Ali M. (Heidelberg: Springer), 295–305. 10.1007/978-3-642-21822-4_30

[B69] Hon N. Poh J.-H. Soon C.-S. (2013). Preoccupied minds feel less control: Sense of agency is modulated by cognitive load. *Conscious. Cogn.* 22 556–561. 10.1016/j.concog.2013.03.004 23584534

[B70] Hon N. Yeo N. (2021). Having a sense of agency can improve memory. *Psychon. Bull. Rev*. 28, 946–952. 10.3758/s13423-020-01849-x 33415660

[B71] Inoue K. Takeda Y. Kimura M. (2017). Sense of agency in continuous action: Assistance-induced performance improvement is self-attributed even with knowledge of assistance. *Conscious. Cogn.* 48 246–252. 10.1016/j.concog.2016.12.003 28027510

[B72] International Civil Aviation Organization (2017). *International civil aviation organization, safety report.* Montreal, QC: International Civil Aviation Organization, 25.

[B73] Jammes Y. Behr M. Llari M. Bonicel S. Weber J. P. Berdah S. (2017). Emergency braking is affected by the use of cruise control. *Traffic Inj. Prev.* 18 636–641. 10.1080/15389588.2016.1274978 28118033

[B74] Jeannerod M. (2003). The mechanism of self-recognition in humans. *Behav. Brain Res.* 142 1–15. 10.1016/S0166-4328(02)00384-4 12798261

[B75] Kaber D. B. Endsley M. R. (1997). Out-of-the-loop performance problems and the use of intermediate levels of automation for improved control system functioning and safety. *Process Safety Prog.* 16 126–131. 10.1002/prs.680160304

[B76] Kawabe T. (2013). Inferring sense of agency from the quantitative aspect of action outcome. *Conscious. Cogn.* 22 407–412. 10.1016/j.concog.2013.01.006 23416540

[B77] Kessel C. J. Wickens C. D. (1982). The transfer of failure-detection skills between monitoring and controlling dynamic systems. *Hum. Fact. J. Hum. Fact. Ergono. Soc.* 24 49–60. 10.1177/001872088202400106

[B78] Khamassi M. Pacherie E. (2018). “L’ACTION,” in *La cognition: du neurone à la société*, eds Collins T. Andler D. Tallon-Baudry C. (Paris: Gallimard).

[B79] Kovács ÁM. Téglás E. Endress A. D. (2010). The social sense: Susceptibility to others’ beliefs in human infants and adults. *Science* 330 1830–1834. 10.1126/science.1190792 21205671

[B80] Kühn S. Nenchev I. Haggard P. Brass M. Gallinat J. Voss M. (2011). Whodunnit? Electrophysiological correlates of agency judgements. *PLoS One* 6:e28657. 10.1371/journal.pone.0028657 22194878 PMC3237473

[B81] Lausic D. Tennebaum G. Eccles D. Jeong A. Johnson T. (2009). Intrateam communication and performance in doubles tennis. *Res. Q. Exerc. Sport* 80 281–290. 10.1080/02701367.2009.10599563 19650394

[B82] Le Bars S. Devaux A. Nevidal T. Chambon V. Pacherie E. (2020). Agents’ pivotality and reward fairness modulate sense of agency in cooperative joint action. *Cognition* 195:104117. 10.1016/j.cognition.2019.104117 31751814

[B83] Le Goff K. Rey A. Berberian B. (2015). “Toward a model for effective human-automation interaction: the mediated agency,” in *Digital human modeling. applications in health, safety, ergonomics and risk management: ergonomics and health*, ed. Duffy V. G. (Cham: Springer International Publishing), 274–283. 10.1007/978-3-319-21070-4_28

[B84] Le Goff K. Rey A. Haggard P. Oullier O. Berberian B. (2018). Agency modulates interactions with automation technologies. *Ergonomics* 61 1282–1297. 29683404 10.1080/00140139.2018.1468493

[B85] Li P. Han C. Lei Y. Holroyd C. B. Li H. (2011). Responsibility modulates neural mechanisms of outcome processing: An ERP study. *Psychophysiology* 48 1129–1133. 10.1111/j.1469-8986.2011.01182.x 21729102

[B86] Libet B. (1985). Unconscious cerebral initiative and the role of conscious will in voluntary action. *Behav. Brain Sci.* 8 529–566. 10.1017/S0140525X00044903

[B87] Libet B. Gleason C. A. Wright E. W. Pearl D. K. (1983). Time of conscious intention to act in relation to onset of cerebral activity (readiness-potential). The unconscious initiation of a freely voluntary act. *Brain:J. Neurol.* 106(Pt 3) 623–642. 10.1093/brain/106.3.623 6640273

[B88] Lucas C. G. Kemp C. (2015). An improved probabilistic account of counterfactual reasoning. *Psychol. Rev.* 122 700–734. 10.1037/a0039655 26437149

[B89] MacKenzie I. S. Ware C. (1993). “Lag as a determinant of human performance in interactive systems,” in *Proceedings of the INTERACT ‘93 and CHI ’93 conference on human factors in computing systems*, (New York, NY: ACM), 488–493. 10.1145/169059.169431

[B90] McCarthy J. Minsky M. L. Rochester N. Shannon C. E. (1955). A proposal for the dartmouth summer research project on artificial intelligence. August 31, 1955 (No. 4). *AI Mag* 27 12–14. 10.1609/aimag.v27i4.1904

[B91] McNeese N. J. Demir M. Cooke N. J. Myers C. (2018). Teaming with a synthetic teammate: insights into human-autonomy teaming. *Hum. Fact.* 60 262–273. 10.1177/0018720817743223 29185818

[B92] Metcalfe J. Eich T. S. Miele D. B. (2013). Metacognition of agency: Proximal action and distal outcome. *Exp. Brain Res.* 229 485–496. 10.1007/s00221-012-3371-6 23358706

[B93] Metcalfe J. Greene M. J. (2007). Metacognition of agency. *J. Exp. Psychol. Gen.* 136 184–199. 10.1037/0096-3445.136.2.184 17500645

[B94] Miller T. (2018). Explanation in artificial intelligence: Insights from the social sciences. *arXiv* [Preprint] 10.48550/arXiv.1706.07269 35895330

[B95] Moore J. W. Fletcher P. C. (2011). Sense of agency in health and disease: A review of cue integration approaches. *Conscious. Cogn.* 21 59–68. 10.1016/j.concog.2011.08.010 21920777 PMC3315009

[B96] Moore J. W. Obhi S. S. (2012). Intentional binding and the sense of agency: A review. *Conscious. Cogn.* 21 546–561. 10.1016/j.concog.2011.12.002 22240158

[B97] Moore J. W. Wegner D. M. Haggard P. (2009). Modulating the sense of agency with external cues. *Conscious. Cogn.* 18 1056–1064. 10.1016/j.concog.2009.05.004 19515577

[B98] Mueller S. T. Veinott E. S. Hoffman R. R. Klein G. Alam L. Mamun T. (2021). Principles of explanation in human-AI systems. *arXiv [Preprint]*. arXiv:2102.04972.

[B99] Mulder M. Abbink D. A. Boer E. R. (2012). Sharing control with haptics: seamless driver support from manual to automatic control. *Hum. Fact.* 54 786–798. 10.1177/0018720812443984 23156623

[B100] Mylopoulos M. Pacherie E. (2019). Intentions: The dynamic hierarchical model revisited. *WIREs Cogn. Sci.* 10:e1481. 10.1002/wcs.1481 30105894

[B101] Mynatt C. Sherman S. J. (1975). Responsibility attribution in groups and individuals: A direct test of the diffusion of responsibility hypothesis. *J. Personal. Soc. Psychol.* 32 1111–1118. 10.1037/0022-3514.32.6.1111

[B102] Nakashima R. Kumada T. (2020). Explicit sense of agency in an automatic control situation: Effects of goal-directed action and the gradual emergence of outcome. *Front. Psychol.* 11:2062. 10.3389/fpsyg.2020.02062 32982855 PMC7488175

[B103] Navarro J. François M. Mars F. (2016). Obstacle avoidance under automated steering: Impact on driving and gaze behaviours. *Transp. Res. Part F Traffic Psychol. Behav.* 43 315–324. 10.1016/j.trf.2016.09.007

[B104] Norman D. A. Broadbent D. E. Baddeley A. D. Reason J. (1990). The ‘problem ‘ with automation: Inappropriate feedback and interaction, not ‘over-automation.’. *Philos. Trans. Royal Soc. Lond. Biol. Sci.* 327 585–593. 10.1098/rstb.1990.0101 1970904

[B105] Oberman L. M. McCleery J. P. Ramachandran V. S. Pineda J. A. (2007). EEG evidence for mirror neuron activity during the observation of human and robot actions: Toward an analysis of the human qualities of interactive robots. *Neurocomputing* 70 2194–2203. 10.1016/j.neucom.2006.02.024

[B106] Obhi S. S. Hall P. (2011). Sense of agency and intentional binding in joint action. *Exp. Brain Res.* 211 655–662. 10.1007/s00221-011-2675-2 21503647

[B107] Ongsulee P. (2017). “Artificial intelligence, machine learning and deep learning,” in *Proceedings of the 2017 15th international conference on ICT and knowledge engineering (ICT&KE)*, Bangkok, 1–6. 10.1109/ICTKE.2017.8259629

[B108] Pacherie E. (2000). The content of intentions. *Mind Lang.* 15 400–432. 10.1111/1468-0017.00142

[B109] Pacherie E. (2007). The sense of control and the sense of agency. *Psyche Interdiscip. J. Res. Conscious.* 13 1–30.

[B110] Pacherie E. (2008). The phenomenology of action: A conceptual framework. *Cognition* 107 179–217. 10.1016/j.cognition.2007.09.003 17950720

[B111] Pacherie E. (2012). “The phenomenology of joint action: self-agency versus joint agency,” in *Joint attention*, ed. Seemann A. (Cambridge, MA: The MIT Press), 10.7551/mitpress/8841.003.0017

[B112] Pacherie E. (2013). Intentional joint agency: Shared intention lite. *Synthese* 190 1817–1839. 10.1007/s11229-013-0263-7

[B113] Pacherie E. (2014). How does it feel to act together? *Phenomenol. Cogn. Sci.* 13 25–46. 10.1007/s11097-013-9329-8

[B114] Pacherie E. (2015). “Time to act: The dynamics of agentive experiences,” in *The sense of agency: social cognition and social neuroscience*, eds Haggard P. Eitam B. (Oxford: Oxford University Press).

[B115] Perez-Marcos D. Slater M. Sanchez-Vives M. V. (2009). Inducing a virtual hand ownership illusion through a brain–computer interface. *NeuroReport* 20 589–594. 10.1097/WNR.0b013e32832a0a2a 19938302

[B116] Perez-Osorio J. Wykowska A. (2020). Adopting the intentional stance toward natural and artificial agents. *Philos. Psychol.* 33 369–395. 10.1080/09515089.2019.1688778

[B117] Poizat G. Bourbousson J. Saury J. Sève C. (2009). Analysis of contextual information sharing during table tennis matches: An empirical study of coordination in sports. *Int. J. Sport Exerc. Psychol.* 7 465–487. 10.1080/1612197X.2009.9671920

[B118] Putnam V. Conati C. (2019). “Exploring the need for explainable artificial intelligence (XAI) in intelligent tutoring systems (ITS),” in *Proceedings of the joint proceedings of the ACM IUI 2019 workshops co-located with the 24th ACM conference on intelligent user interfaces, ACM*, (Los Angeles, CA).

[B119] Robbins S. (2019). A misdirected principle with a catch: Explicability for AI. *Minds Mach.* 29 495–514. 10.1007/s11023-019-09509-3

[B120] Sahaï A. Desantis A. Grynszpan O. Pacherie E. Berberian B. (2019). Action co-representation and the sense of agency during a joint Simon task: Comparing human and machine co-agents. *Conscious. Cogn.* 67 44–55. 10.1016/j.concog.2018.11.008 30522081

[B121] Sahai A. Pacherie E. Grynszpan O. Berberian B. (2017). “Co-representation of human-generated actions vs. machine-generated actions: Impact on our sense of we-agency?,” in *Proceedings of the 2017 26th IEEE international symposium on robot and human interactive communication (RO-MAN)*, (Lisbon), 341–345. 10.1109/ROMAN.2017.8172324

[B122] Samson D. Apperly I. A. Braithwaite J. J. Andrews B. J. Bodley Scott S. E. (2010). Seeing it their way: Evidence for rapid and involuntary computation of what other people see. *J. Exp. Psychol. Hum. Percep. Perform.* 36 1255–1266. 10.1037/a0018729 20731512

[B123] San Martín R. (2012). Event-related potential studies of outcome processing and feedback-guided learning. *Front. Hum. Neurosci.* 6:304. 10.3389/fnhum.2012.00304 23162451 PMC3491353

[B124] Santoni de Sio F. van den Hoven J. (2018). Meaningful human control over autonomous systems: A philosophical account. *Front. Robot. AI* 5:15. 10.3389/frobt.2018.00015 33500902 PMC7806098

[B125] Sarter N. B. Woods D. D. (1995). How in the world did we ever get into that mode? Mode error and awareness in supervisory control. *Hum. Fact.* 37 5–19. 10.1518/001872095779049516

[B126] Sarter N. B. Woods D. D. Billings C. E. (1997). “Automation surprises,” in *Handbook of human factors and ergonomics*, 2nd Edn, ed. Salvendy G. (New York, NY: Wiley), 1926–1943.

[B127] Sato A. Yasuda A. (2005). Illusion of sense of self-agency: Discrepancy between the predicted and actual sensory consequences of actions modulates the sense of self-agency, but not the sense of self-ownership. *Cognition* 94 241–255. 10.1016/j.cognition.2004.04.003 15617673

[B128] Sebanz N. Bekkering H. Knoblich G. (2006). Joint action: Bodies and minds moving together. *Trends Cogn. Sci.* 10 70–76. 10.1016/j.tics.2005.12.009 16406326

[B129] Sebanz N. Knoblich G. Prinz W. (2003). Representing others’ actions: Just like one’s own? *Cognition* 88 B11–B21. 10.1016/S0010-0277(03)00043-X 12804818

[B130] Sebanz N. Knoblich G. Prinz W. (2005). How two share a task: Corepresenting stimulus-response mappings. *J. Exp. Psychol. Hum. Percep. Perform.* 31 1234–1246. 10.1037/0096-1523.31.6.1234 16366786

[B131] Shekhar S. S. (2019). Artificial intelligence in automation. *Artif. Intell*. 3085, 14–17.

[B132] Sheridan T. B. (2002). *Humans and automation: System design and research issues (pp. xii, 264). human factors and ergonomics society.* Santa Barbara, CA: Cambridge University Press.

[B133] Sheridan T. B. Verplank W. L. (1978). *Human and computer control of undersea teleoperators.* Fort Belvoir, VA: Defense Technical Information Center, 10.21236/ADA057655

[B134] Shneiderman B. Plaisant C. (2004). *Designing the user interface.* London: Pearson.

[B135] Sidarus N. Chambon V. Haggard P. (2013). Priming of actions increases sense of control over unexpected outcomes. *Conscious. Cogn.* 22 1403–1411. 10.1016/j.concog.2013.09.008 24185190

[B136] Sidarus N. Haggard P. (2016). Difficult action decisions reduce the sense of agency: A study using the Eriksen flanker task. *Acta Psychol.* 166 1–11. 10.1016/j.actpsy.2016.03.003 27017411

[B137] Sidarus N. Palminteri S. Chambon V. (2019). Cost-benefit trade-offs in decision-making and learning. *PLoS Comput. Biol.* 15:e1007326. 10.1371/journal.pcbi.1007326 31490934 PMC6750595

[B138] Sidarus N. Vuorre M. Metcalfe J. Haggard P. (2017). Investigating the prospective sense of agency: effects of processing fluency, stimulus ambiguity, and response conflict. *Front. Psychol.* 8:545. 10.3389/fpsyg.2017.00545 28450839 PMC5389984

[B139] Silver C. A. Tatler B. W. Chakravarthi R. Timmermans B. (2020). Social agency as a continuum. *Psycho. Bull. Rev.* 28 434–453. 10.3758/s13423-020-01845-1 33289061 PMC8062427

[B140] Slater M. Pérez Marcos D. Ehrsson H. Sanchez-Vives M. V. (2009). Inducing illusory ownership of a virtual body. *Front. Neurosci.* 3:214–220. 10.3389/neuro.01.029.2009 20011144 PMC2751618

[B141] Tanimoto T. Shinohara K. Yoshinada H. (2017). Research on effective teleoperation of construction machinery fusing manual and automatic operation. *Robomech J.* 4:14. 10.1186/s40648-017-0083-5

[B142] Thellman S. Silvervarg A. Ziemke T. (2017). Folk-psychological interpretation of human vs. humanoid robot behavior: Exploring the intentional stance toward robots. *Front. Psychol.* 8:1962. 10.3389/fpsyg.2017.01962 29184519 PMC5694477

[B143] Timm J. SanMiguel I. Keil J. Schröger E. Schönwiesner M. (2014). Motor intention determines sensory attenuation of brain responses to self-initiated sounds. *J. Cogn. Neurosci.* 26 1481–1489. 10.1162/jocn_a_00552 24392902

[B144] Tintarev N. Masthoff J. (2010). “Designing and evaluating explanations for recommender systems,” in *Recommender systems handbook*, eds Ricci F. Rokach L. Shapira B. Kantor P. B. (New York, NY: Springer), 479–510. 10.1007/978-0-387-85820-3

[B145] Tintarev N. Masthoff J. (2012). Evaluating the effectiveness of explanations for recommender systems: Methodological issues and empirical studies on the impact of personalization. *User Model. UserAdapt. Inter.* 22 399–439. 10.1007/s11257-011-9117-5

[B146] Tintarev N. Masthoff J. (2015). “Explaining recommendations: design and evaluation,” in *Recommender systems handbook*, eds Ricci F. Rokach L. Shapira B. (New York, NY: Springer US), 353-382. 10.1007/978-1-4899-7637-6_10

[B147] Ueda S. Nakashima R. Kumada T. (2021). Influence of levels of automation on the sense of agency during continuous action. *Sci. Rep.* 11:2436. 10.1038/s41598-021-82036-3 33510395 PMC7843606

[B148] Van Der Laan J. D. Heino A. De Waard D. (1997). A simple procedure for the assessment of acceptance of advanced transport telematics. *Transp. Res. Part C Emerg. Technol.* 5 1–10. 10.1016/S0968-090X(96)00025-3

[B149] van der Wel R. P. R. D. (2015). Me and we: Metacognition and performance evaluation of joint actions. *Cognition* 140 49–59. 10.1016/j.cognition.2015.03.011 25880341

[B150] van der Wel R. P. R. D. Sebanz N. Knoblich G. (2012). The sense of agency during skill learning in individuals and dyads. *Conscious. Cogn.* 21 1267–1279. 10.1016/j.concog.2012.04.001 22541646

[B151] van Hateren J. H. (2015). The origin of agency, consciousness, and free will. *Phenomenol. Cogn. Sci.* 14 979–1000. 10.1007/s11097-014-9396-5

[B152] van Lent M. Fisher W. Mancuso M. (2004). “An explainable artificial intelligence system for small-unit tactical behavior,” in *Proceedings of the 16th conference. on innovative applications of artificial intelligence*, (San Jose, CA: AAAI Press).

[B153] Vantrepotte Q. Berberian B. Pagliari M. Chambon V. (2022). Leveraging human agency to improve confidence and acceptability in human-machine interactions. *Cognition* 222:105020. 10.1016/j.cognition.2022.105020 35033865

[B154] Victor T. W. Tivesten E. Gustavsson P. Johansson J. Sangberg F. Ljung Aust M. (2018). Automation expectation mismatch: incorrect prediction despite eyes on threat and hands on wheel. *Hum. Fact.* 60 1095–1116. 10.1177/0018720818788164 30096002 PMC6207994

[B155] Vinding M. C. Pedersen M. N. Overgaard M. (2013). Unravelling intention: Distal intentions increase the subjective sense of agency. *Conscious. Cogn.* 22 810–815. 10.1016/j.concog.2013.05.003 23732190

[B156] Voss M. Moore J. Hauser M. Gallinat J. Heinz A. Haggard P. (2010). Altered awareness of action in schizophrenia: A specific deficit in predicting action consequences. *Brain* 133 3104–3112. 10.1093/brain/awq152 20685805

[B157] Wachter S. Mittelstadt B. Russell C. (2018). Counterfactual explanations without opening the black box: Automated decisions and the GDPR. *Harv. J. Law Technol.* 31 841–887.

[B158] Wahn B. Kingstone A. König P. (2018). Group benefits in joint perceptual tasks-a review: Group benefits in joint perceptual tasks. *Ann. N. Y. Acad. Sci.* 1426 166–178. 10.1111/nyas.13843 29754443

[B159] Wang Z. Zheng R. Kaizuka T. Nakano K. (2019). Relationship between gaze behavior and steering performance for driver–automation shared control: a driving simulator study. *IEEE Trans. Intellig. Vehicl.* 4 154–166. 10.1109/TIV.2018.2886654

[B160] Wegner D. M. Sparrow B. Winerman L. (2004). Vicarious agency: Experiencing control over the movements of others. *J. Personal. Soc. Psychol.* 86 838–848. 10.1037/0022-3514.86.6.838 15149258

[B161] Wegner D. M. Wheatley T. (1999). Apparent mental causation. Sources of the experience of will. *Am. Psychol.* 54 480–492. 10.1037//0003-066x.54.7.480 10424155

[B162] Wen W. (2019). Does delay in feedback diminish sense of agency? A review. *Conscious. Cogn.* 73:102759. 10.1016/j.concog.2019.05.007 31173998

[B163] Wen W. Haggard P. (2018). Control changes the way we look at the world. *J. Cogn. Neurosci.* 30 603–619. 10.1162/jocn_a_01226 29308984

[B164] Wen W. Kuroki Y. Asama H. (2019). The sense of agency in driving automation. *Front. Psychol.* 10:2691. 10.3389/fpsyg.2019.02691 31849787 PMC6901395

[B165] Wen W. Yamashita A. Asama H. (2015a). The sense of agency during continuous action: performance is more important than action-feedback association. *PLoS One* 10:e0125226. 10.1371/journal.pone.0125226 25893992 PMC4404253

[B166] Wen W. Yamashita A. Asama H. (2015b). The influence of action-outcome delay and arousal on sense of agency and the intentional binding effect. *Conscious. Cogn.* 36 87–95. 10.1016/j.concog.2015.06.004 26100602

[B167] Wen W. Yun S. Yamashita A. Northcutt B. D. Asama H. (2021). Deceleration assistance mitigated the trade-off between sense of agency and driving performance. *Front. Psychol.* 12:643516. 10.3389/fpsyg.2021.643516 34149526 PMC8208475

[B168] Wenke D. Fleming S. M. Haggard P. (2010). Subliminal priming of actions influences sense of control over effects of action. *Cognition* 115 26–38. 10.1016/j.cognition.2009.10.016 19945697

[B169] Woods D. D. Tinapple D. (1999). “W3: Watching human factors watch people at work presidential address,” in *Proceedings of the presented at the 43rd Annual meeting of the human factors and ergonomics society*, (Houston, TX: Multimedia Production).

[B170] Wykowska A. Chellali R. Al-Amin M. D. M. Müller H. J. (2014). Implications of robot actions for human perception. how do we represent actions of the observed robots? *Int. J. Soc. Robot.* 6 357–366. 10.1007/s12369-014-0239-x

[B171] Yun S. Wen W. An Q. Hamasaki S. Yamakawa H. Tamura Y. (2018). “Investigating the relationship between assisted driver’s SoA and EEG,” in *Converging clinical and engineering research on neurorehabilitation III*, eds Masia L. Micera S. Akay M. Pons J. L. (Berlin: Springer International Publishing), 1039–1043. 10.1007/978-3-030-01845-0_208

[B172] Zanatto D. Chattington M. Noyes J. (2021). “Sense of agency in human-machine interaction,” in *Advances in neuroergonomics and cognitive engineering*, eds Ayaz H. Asgher U. Paletta L. (Cham: Springer International Publishing), 353–360. 10.1007/978-3-030-80285-1_41

